# SNF5 Is an Essential Executor of Epigenetic Regulation during Differentiation

**DOI:** 10.1371/journal.pgen.1003459

**Published:** 2013-04-25

**Authors:** Jueng Soo You, Daniel D. De Carvalho, Chao Dai, Minmin Liu, Kurinji Pandiyan, Xianghong J. Zhou, Gangning Liang, Peter A. Jones

**Affiliations:** 1 Departments of Urology and Biochemistry and Molecular Biology, USC Norris Comprehensive Cancer Center, Keck School of Medicine, University of Southern California, Los Angles, California, United States of America; 2 Department of Biochemistry, Graduate School of Medicine, Konkuk University, Seoul, Republic of Korea; 3 Program in Molecular and Computational Biology, University of Southern California, Los Angles, California, United States of America; 4 Program in Human Genetics, Johns Hopkins University School of Medicine, Baltimore, Maryland, United States of America; University of California San Francisco, United States of America

## Abstract

Nucleosome occupancy controls the accessibility of the transcription machinery to DNA regulatory regions and serves an instructive role for gene expression. Chromatin remodelers, such as the BAF complexes, are responsible for establishing nucleosome occupancy patterns, which are key to epigenetic regulation along with DNA methylation and histone modifications. Some reports have assessed the roles of the BAF complex subunits and stemness in murine embryonic stem cells. However, the details of the relationships between remodelers and transcription factors in altering chromatin configuration, which ultimately affects gene expression during cell differentiation, remain unclear. Here for the first time we demonstrate that SNF5, a core subunit of the BAF complex, negatively regulates OCT4 levels in pluripotent cells and is essential for cell survival during differentiation. SNF5 is responsible for generating nucleosome-depleted regions (NDRs) at the regulatory sites of OCT4 repressed target genes such as PAX6 and NEUROG1, which are crucial for cell fate determination. Concurrently, SNF5 closes the NDRs at the regulatory regions of OCT4-activated target genes such as OCT4 itself and NANOG. Furthermore, using loss- and gain-of-function experiments followed by extensive genome-wide analyses including gene expression microarrays and ChIP-sequencing, we highlight that SNF5 plays dual roles during differentiation by antagonizing the expression of genes that were either activated or repressed by OCT4, respectively. Together, we demonstrate that SNF5 executes the switch between pluripotency and differentiation.

## Author Summary

DNA is packaged with proteins into higher-order chromatin structures, which makes genes inherently resistant to transcription initiation. The importance of chromatin remodelers in inducing structural changes to chromatin and, therefore, in controlling the expression of genes has recently resurfaced with the realization that several of them are mutated in human cancers. SNF5, which serves as the core subunit of the BAF remodeling complex, is one such remodeler. In this study, we identify the role of SNF5 induced chromatin remodeling in cell differentiation, the commitment of embryonic cells to a mature lineage-committed state. Importantly, we find that SNF5 establishes appropriate chromatin remodeling patterns during differentiation by controlling the levels of the OCT4 protein, the master determinant of the undifferentiated state. On receipt of differentiation cues, SNF5 opens the chromatin of repressed genes that are occupied by OCT4. SNF5 also induces the closing of genes that are being actively transcribed and OCT4 bound. Further, we show that SNF5 is necessary for cell survival during differentiation, highlighting its crucial role in the process. Together, our data shed novel insights on the importance of SNF5 in maintaining the balance between the embryonic and differentiated states.

## Introduction

During development, each cell acquires the appropriate gene expression that ensures its cellular identity, which is accomplished by multiple layers of epigenetic regulations [1], [2]. The interactions among transcription factors, covalent chromatin marks, histone variants and chromatin remodelers are essential for the epigenetic signature and ultimate gene expression of cells [3]. Recent extenstive genome wide studies have highlighted the importance of chromatin accessibility associated with transcription factor binding [4], [5]. Pluripotent cells have unique transcriptional circuitries and epigenetic landscapes that allow them to self-renew, yet remain poised to differentiate into each of the three germ layers in response to developmental signals [1], [2], [6]. OCT4, which is the most well-known transcription factor among the core transcriptional regulators, has been implicated in establishing and maintaining pluripotency along with SOX2 and NANOG [7]. These transcription factors work together to positively regulate their own genomic regulatory regions, establishing autoregulatory loops that are critical for the maintenance of pluripotency and the generation of induced pluripotent stem (iPS) cells [1]. OCT4 represses genes involved in cell lineage specification which are frequently Polycomb Group targets and are held in a poised state for activation upon differentiation [7]. Until recently, OCT4 remained the only factor shown to be essential for the generation of iPS cells [8], suggesting that OCT4 is critical to the stem cell signature. The spectrum of OCT4 interaction partners has been explored and shown to be critical for establishing and maintaining pluripotency [9], [10], [11]; however, how the epigenetic regulators associate with OCT4 and its target genes remains largely unknown.

Nucleosomes are the basic building blocks of chromatin and function in packaging and controlling DNA accessibility. Nucleosome positioning serves an instructive role for the transcriptional machinery, directing it to the correct regulatory regions and occluding functional sites [12], [13]. It has been known for several years that nucleosome occupancy itself can regulate gene expression; however, most studies have been focused on the role of covalent modifications associated with nucleosomes. Despite a resurgence of interest in nucleosomes as epigenetic regulators [14], [15], [16], [17], [18], their role is still often overlooked. We have developed a methodology called NOMe-seq (nucleosome occupancy and methylome sequencing), a method that allows us to determine nucleosome occupancy and DNA methylation on the same DNA modules, regardless of CpG density or the endogenous DNA methylation state [19]. Compared to traditional nucleosome positioning methods such as MNase-seq or Histone3 ChIP-seq [20], which rely on DNA breakage, NOMe-seq signal is interpreted as a percentage of sequencing reads at a given position and provides a normalized and unskewed measurement. Furthermore, it can measure subtle nucleosome depletion and NDR size, which is underestimated by previous methods by sonication. Using NOMe-seq, we have shown the central role of the nucleosome depleted regions (NDRs) in various cellular contexts [19], [21], [22], [23].

ATP-dependent chromatin remodelers together with histone modifying complexes control nucleosome occupancy and chromatin structure [24], [25], and are critical players in several biological pathways, such as those involved in cancer, differentiation, immune response, stemness and reprogramming [25], [26], [27], [28], [29], [30], [31]. The importance of these remodeling machines is becoming apparent with the realization that many of their components are mutated in human cancers [32], [33], [34]. Brahma-associated factor (BAF) complexes belong to the SWI/SNF family and constitute a multi-subunit complex, which contain a central ATPase (BRG1 or BRM), core subunits (SNF5, BAF155, BAF170), and accessory subunits [24], [25]. Their subunit composition differ depending on the cellular context [24], [25], for instance, mouse embryonic stem cells have a complex called esBAF, which contains BRG1 and BAF155 instead of BRM and BAF170 [25]. In addition to various compositions, it is also reported that the modification of the complex modulates its activity in certain cellular contexts [35], [36]

SNF5 is one of the core subunits of the BAF complex and its expression levels remain unchanged during cell differentiation unlike BRG1 or other core subunits (BAF155 or BAF170) [25]. SNF5 is also known as a bona fide tumor suppressor based on genetic evidence that the majority of rhabdoid tumors contain bi-allelic inactivating mutations in the *SNF5 (SMARCB1)* locus [37]. A link between SNF5 deregulation and tumorigenesis has been highlighted in broad cancer related target pathways [32], including Hedgehog-Gli [38], Rb and p53 [39], [40]. In addition, epigenetic antagonism between the polycomb repressor complex subunit EZH2 and SNF5 has been reported during oncogenic transformation [41]. Polycomb repressor complexes are highly active in embryonic stem cells and since OCT4, the master regulator of pluripotency, targets polycomb genes [1], [7], determining the relationship between SNF5 and OCT4 may help elucidate the epigenetic networks involved in the regulation of gene expression in pluripotent cells. Although some studies have shown that BAF complexes are important for maintaining the pluripotent state of mouse embryonic stem cells [26], [42], [43], [44], [45], the relationship between SNF5 and OCT4 remains to be studied.

In this study, we demonstrate that SNF5 plays a previously overlooked role as an essential epigenetic regulator during human pluripotent cell differentiation. Our results indicate that SNF5 acts to fine-tune OCT4 levels in the pluripotent state. On the other hand, during differentiation SNF5 plays the dual role of repressing OCT4 activated genes and activating OCT4 repressed genes, thereby determining cell fates. Using loss and gain of function experiments, we show that SNF5 knockdown or overexpression disrupts the balance between pluripotency and differentiation by changing nucleosome occupancy at the regulatory regions of OCT4 target genes, thus affecting their transcriptional state. Loss of SNF5 during differentiation leads to cell death, indicating that SNF5 is required for cell survival during differentiation. In contrast, gain of SNF5 induces premature differentiation by antagonizing OCT4 pluripotency functions. Genome-wide expression analyses and SNF5 ChIP-sequencing further support the concept that SNF5 modulates OCT4 target genes and controls cell differentiation potential and survival. Taken together, the results presented here highlight the important role of SNF5 in the networks controlling the balance between pluripotency and differentiation.

## Results

### OCT4 target genes show distinctive nucleosome occupancy patterns that underlie the potential for gene expression

Previously, we demonstrated that the regulatory regions of OCT4 activated genes such as the *OCT4* distal enhancer (DE) and *NANOG* proximal promoter (PP) are nucleosome depleted in pluripotent cells and bound by OCT4. OCT4 binding to these regulatory regions is important for establishing NDRs and these regions become nucleosome occupied and DNA methylated during differentiation [19].

**Figure 1:**
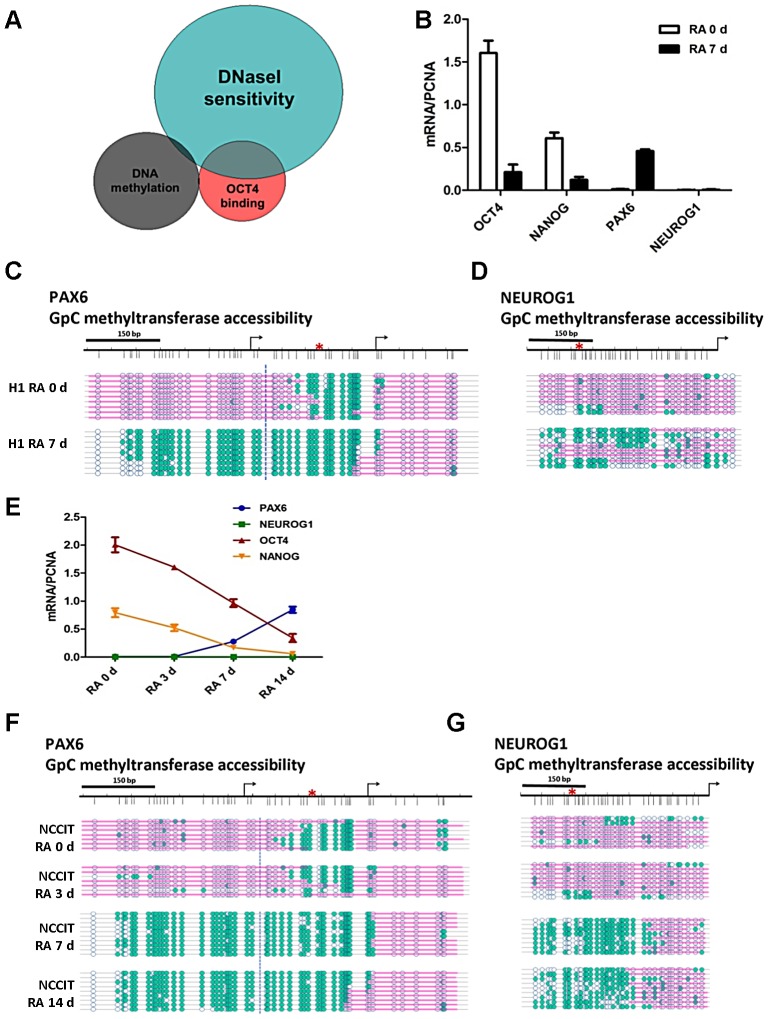
OCT4 target genes show distinctive nucleosome occupancy patterns that underlie the potential for gene expression. (A) Genome-wide studies were performed in human embryonic stem cells (H1) using ENCODE and GEO data (wgEncodeHudsonalphaMethylSeqRegionsRep1H1hesc for DNA methylation, GSM518373 for OCT4 ChIP-Seq and wgEncodeUwDnaseSeqPeaksRep1H1es for DNaseI). The data comprised 100 bp windows of OCT4 binding regions (29740 sites), DNA methylated regions (43659 sites) and DNaseI hypersensitive regions (123778 sites). (B and E) H1 and NCCIT cells were exposed to 10 uM RA for the indicated days. The expression levels of OCT4, NANOG, PAX6 and NEUROG1 were determined by quantitative PCR (normalized to PCNA). Quantitative PCR data represent the average of three biological experiments (the mean +SEM) (C, D, F and G) Nucleosome occupancy at the *PAX6* and *NEUROG1* promoters was analyzed by NOMe-seq during differentiation of H1 and NCCIT cells. Blue circles represent GpC sites of the DNA (unfilled blue circles represent GpC sites which are inaccessible to GpC methyltransferase, teal-filled circles represent cytosines accessible to GpC methyltransferase). Pink bars represent regions of inaccessibility large enough to accommodate a nucleosome (around 150 bp). The data is representative of three biological experiments.

To gain a further understanding of the role of OCT4 in controlling nucleosome occupancy on a genome-wide scale, we performed an in-silico comparison of DNaseI hypersensitive, DNA methylation and OCT4 bound regions for the human embryonic stem cell line H1 using publically available data. Results from these studies show that, in H1 cells, 46% of OCT4 bound sites DNaseI are hypersensitive, whereas 64% of OCT4 bound regions are DNaseI resistant (Figure 1A). This suggests that OCT4 has the ability to associate with different chromatin structures, which is in agreement with its proposed dual role as a transcriptional activator and repressor [7]. From this analysis we found that OCT4 activated genes such as *OCT4* and *NANOG* display DNaseI hypersensitivity, suggesting that they have a more open chromatin structure, whereas genes known to be repressed by OCT4 such as *PAX6* and *NEUROG1* are DNaseI resistant, suggesting a closed structure (Figure 1A). Notably, the DNaseI resistant group is enriched for polycomb-repressed genes in embryonic stem cells (p-value 3.41×10^−9^).

To study nucleosome occupancy changes at genes that are either activated or repressed by OCT4 during pluripotent cell differentiation, we first studied changes in gene expression and nucleosome occupancy as a function of differentiation of the human pluripotent embryonic stem cell line H1. We treated H1 with 10 uM retinoic acid (RA) for up to 7 days to induce cell differentiation. As expected, we observed decreased mRNA expression of OCT4, NANOG and SOX2 after RA treatment (Figure 1B) and the cells underwent morphological changes characteristic of differentiated cells (a decrease in the nuclear/cytoplasmic ratio; data not shown). Since RA can skew the differentiation of the cells toward the neuro/ectodermal lineage, we selected *PAX6* and *NEUROG1*, important players in neuro/ectoderm differentiation [46], [47], for in depth study. We observed the increased expression of PAX6 on RA exposure, however RA did not induce NEUROG1 expression in H1 cells even after 7 days of treatment (Figure 1B), suggesting that additional factors might be needed for NEUROG1 expression. To understand the changes in nucleosome occupancy and DNA methylation that influence the gene expression patterns during differentiation, we performed NOMe-seq. The regulatory regions of the OCT4 activated targets *OCT4* and *NANOG* are nucleosome depleted [19] in human pluripotent cells, in agreement with the genome-wide data shown in Figure 1A. NOMe-seq results for undifferentiated H1 cells showed that the promoters of the OCT4 repressed target genes *PAX6* and *NEUROG1* are unmethylated yet largely nucleosome occupied (Figure 1C, 1D and S1B). Interestingly, the *PAX6* promoter showed a unique NDR at the OCT4 binding site, not seen in the *NEUROG1* promoter in the pluripotent state (Figure 1C and 1D). The ∼500 bp NDR generated at the *PAX6* promoter is large enough to accommodate two to three nucleosomes (Figure 1C), which was also confirmed in another embryonic stem cell line H9 (Figure S1C). Upon induction of cell differentiation, the *NEUROG1* promoter began to open; however, it retained a nucleosome positioned upstream of the transcription start site (TSS) (Figure 1D), correlating with the lack of expression (Figure 1B). Indeed, the nucleosome just upstream of the NEUROG1 was absent in RA treated H9 cells and in glioblastoma 248 cells which express NEUROG1 (Figure S1D and S1E), suggesting that the NDR right upstream of TSS is important for gene expression. Nucleosome occupancy changes after RA treatment were also observed at the promoter region of other genes activated or repressed by OCT4, including *SOX2* (no NDR), and the master regulators of the three germ layers *CDX2* (trophectoderm, NDR), *TBX3 (*mesoderm, NDR) and *ONECUT1* (endoderm, NDR); as shown before, nucleosome occupancy correlated with gene expression patterns (Figure S1F and S1G). More detailed studies performed with the NCCIT cells, which have a comparable transcriptional profile of H1 [48], showed essentially the same results (Figure 1E, 1F and 1G). Together, these data show that OCT4 target genes have unique nucleosome occupancy patterns underlying the potential for gene expression. Furthermore, these nucleosome configurations change substantially during differentiation, which allows for the establishment of new transcriptional states.

### SNF5 is recruited to OCT4-activated and -repressed genes with distinctive chromatin landscapes during differentiation

**Figure 2:**
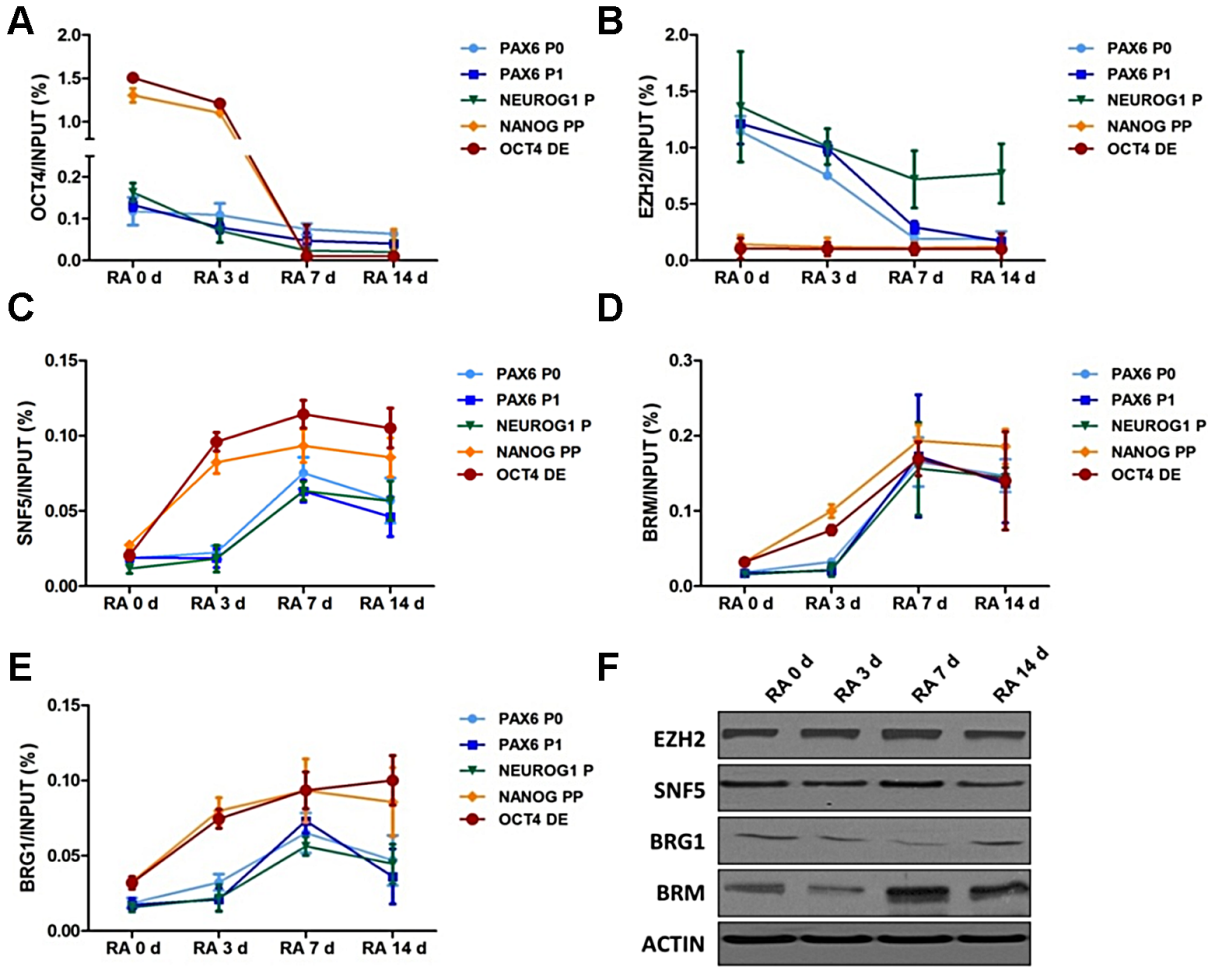
SNF5 is recruited to OCT4-activated and -repressed genes with distinctive chromatin landscape during differentiation. (A–E) Chromatin from NCCIT cells was immunoprecipitated with anti-OCT4 (A), anti-EZH2 (B), anti-SNF5 (C), anti-BRM (D), anti-BRG1 (E) or anti-H3 antibodies and their binding at the DNA regulatory regions of OCT4 target genes were analyzed by quantitative PCR. Quantitative PCR data represent the average of three biological experiments (the mean +SEM). A Mann-Whitney test was performed and the increase in recruitment of SNF5, BRM and BRG1 at OCT4 target genes during differentiation as found to be statistically significant with p-values of 0.013 (SNF5), 0.012 (BRM) and 0.029 (BRG1). (F) The protein level of EZH2, SNF5, BRG1, BRM and loading control ACTIN were subsequently analyzed by western blot. The data is representative of three biological experiments.

To define the chromatin landscape that characterizes OCT4 target genes during cell differentiation, we performed chromatin immunoprecipitation (ChIP) assays using antibodies against the transcription factor OCT4, RNA polymerase II (Pol2), and both active (H3K4me3) and repressive (H3K27me3) epigenetic marks [49] in NCCIT cells (Figure 2A and Figure S2). First, we measured OCT4 binding to the *OCT4* DE, the *NANOG* PP, and the *PAX6* and *NEUROG1* promoters. We found that OCT4 binding decreases upon induction of RA-induced cell differentiation and that regulatory regions of OCT4 activated genes *OCT4* and *NANOG* showed a tenfold enrichment in OCT4 binding compared to those of the repressed genes *PAX6* and *NEUROG1* (Figure 2A). On induction of differentiation, OCT4 activated regions *OCT4* DE and *NANOG* PP showed decreases in the active H3K4me3 and minor increases in the repressive H3K27me3 marks (Figure S2A and S2B). OCT4 repressed regions changed from a bivalent to a monovalent H3K4me3 state at the *PAX6* promoter, while the *NEUROG1* promoter retained the bivalent H3K4me3/H3K27me3 state. The *GAPDH* promoter region was included as a negative control for H3K27me3 and as a positive control for H3K4me3 binding (Figure S2A and S2B). In addition, enrichment in acetylated H3 (AcH3), a marker of open chromatin, positively correlated with the expression of OCT4 target genes (Figure S2C). The levels of all histone marks were normalized to H3 levels to consider nucleosome density (Figure S2D). Interestingly, in the pluripotent state, the bivalent *PAX6* and *NEUROG1* promoters showed some degree of Pol2 occupancy despite the lack of active transcription from these sites (Figure S2E). Throughout cell differentiation, Pol2 binding increased only at the *PAX6* promoter, which correlated with its increased transcription (Figure S2E).

To determine how these chromatin structures are established, we next examined the recruitment of the H3K27 methyltransferase EZH2 [50] and the BAF core subunit SNF5 in response to RA treatment. EZH2 binding showed a distribution pattern similar to that of H3K27me3, while SNF5 enrichment increased over time at all target regions (Figure 2B and 2C). Notably, the kinetics of SNF5 binding were different for OCT4 activated (*OCT4* and *NANOG*) and repressed genes (*PAX6* and *NEUROG1*). The *OCT4* DE and *NANOG* PP showed early SNF5 enrichment, starting three days after RA treatment, which correlates well with the increased nucleosome occupancy at these regions (Figure 2C and Figure 1E). In contrast, the *PAX6* and *NEUROG1* promoters showed a delayed recruitment of SNF5, with levels peaking at seven days after RA treatment, which correlated with nucleosome depletion at these regions (Figure 2C and Figure 1E), suggesting that SNF5 is likely to be responsible nucleosome occupancy changes of these genes. An increase in SNF5 enrichment was also observed after RA-induced differentiation at other OCT4 regulated regions, including the *SOX2*, *CDX2*, *TBX3* and *ONECUT1* promoters (Figure S2F).

To determine whether SNF5 enrichment parallels the recruitment of the BAF complex, we performed ChIP assays for other subunits including BRG1 and BRM, which are the ATPase subunits of the complex, and another core subunit BAF170 (Figure 2D, 2E and Figure S2G). The binding of BRM and BAF170 was increased at all queried regions during differentiation and was accompanied by an increase in protein levels during this process (Figure 2D, Figure S2G and Figure 2F). Despite a decrease in BRG1 protein level during differentiation, BRG1 showed increased enrichment at the above regions (Figure 2E and 2F). Thus, BRG1, BRM and BAF170 enrichment at the promoter regions analyzed here mirror that of SNF5. This suggests that the BAF complex, including SNF5, plays an important role at OCT4 activated and repressed target genes by altering nucleosome occupancy during differentiation. This orchestration of transcription factors, epigenetic marks and chromatin remodelers determines the chromatin landscape at the regulatory regions of OCT4 target genes and underlies their transcriptional potential.

### Knockdown of SNF5 enhances a stem cell like state and blocks differentiation

**Figure 3:**
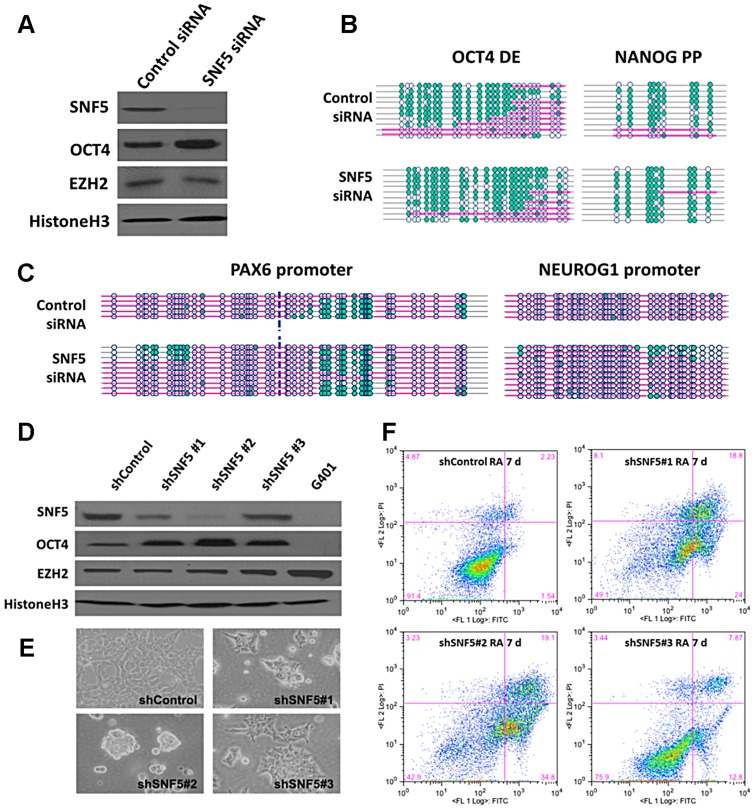
Knockdown of SNF5 enhances a stem cell like state and blocks differentiation. (A) SNF5, OCT4, EZH2, and loading control histone H3 were analyzed by western blot, 72 h post-transfection with SNF5 siRNA in NCCIT cells. (B and C) To get the nucleosome footprint, we have performed at least three biological replicates of NOMe-seq at 72 h post-transfection with SNF5 siRNA in NCCIT and selected ∼10 sequences in an unbiased manner to represented in the figures. (D) Stably infected SNF5 knockdown NCCIT cells were selected for 21 days with antibiotics and SNF5, OCT4, EZH2, and loading control histone H3 were subsequently analyzed by western blot. G401 cells were used for a SNF5 knockdown control. (E) At the same time point, cell morphology micrographs (200X) were taken. The data is representative of three biological experiments. (F) Apoptosis of SNF5 knockdown NCCIT cells after RA treatment was determined by flow cytometric analysis. The X axis indicates Annexin V and the Y axis indicates Propidium iodide (PI). The data are representative of three biological experiments (the mean +SEM).

To examine whether SNF5 is required for the nucleosome occupancy changes observed at OCT4 target genes during differentiation, we knocked it down using targeted siRNAs (Figure 3A). Surprisingly, we detected an increase in OCT4 and NANOG levels after knockdown of SNF5 but little change in those of PAX6 and NEUROG1 (Figure 3A and Figure S3A). Next, we determined whether SNF5 knockdown has an effect on nucleosome occupancy at OCT4 activated and repressed target genes. We found a slight increase in nucleosome depletion at both *OCT4* DE (from 62% to 83%) and *NANOG* PP (from 84% to 97%), which was associated with an increase in expression (Figure 3B and Figure S3A). The *PAX6* and *NEUROG1* promoters did not show changes in nucleosome occupancy correlating with the minimal changes observed in gene expression (Figure 3C and Figure S3A). Similar to the SNF5 knockdown, a partial knockdown of BRG1 (∼55%) resulted in a minor increase in OCT4 (∼30%) and NANOG (∼50%) protein levels (Figure S3B), which correlated with the changes observed in nucleosome occupancy at those regions (Figure S3C).

We then generated stable SNF5 knockdown cells using three different shRNAs for SNF5 to study the prolonged effects of the absence of SNF5 in pluripotent cells and to test the role of SNF5 during differentiation. We analyzed the expression levels of SNF5, OCT4, EZH2 and OCT4 target genes and determined that shRNA #2 was the most efficient, resulting in SNF5 levels comparable to those found in SNF5 null G401 cells (Figure 3D and Figure S3D). OCT4 levels showed a remarkable anti-correlation with SNF5 levels, whereas EZH2 levels remained unchanged, irrespective of SNF5 levels (Figure 3D). Interestingly, NCCIT cells sustained dramatic changes in cell morphology, appearing less flattened and forming sphere-like clusters after stable SNF5 knockdown (Figure 3E), suggesting that SNF5 is critical for maintaining the cellular identity.

Next, we treated all three stable cell lines with RA to induce differentiation and found that SNF5 knockdown cell lines underwent massive cell death compared to those cells infected with a control shRNA, demonstrating that SNF5 is essential for survival during the process of differentiation (Figure 3F). We tried to knock down SNF5 in differentiated cells already treated with RA for 7 days. However, the cells could not survive in the absence of SNF5 (data not shown), suggesting that SNF5 is also critical for the survival of differentiated cells. To ensure that these changes are not specific to NCCIT cells, we also knocked down SNF5 in H1 cells using shRNA#2 (Figure S3E). Although the generation of stable knockdown H1 cells was technically challenging due to their low transduction efficiency, we were able to validate the increase in OCT4 and NANOG levels after SNF5 knockdown in these cells (Figure S3E). Altogether, these data demonstrate that SNF5 is essential for cell survival during differentiation and it plays a crucial role in maintaining the fine balance between pluripotency and differentiation via the regulation of nucleosome occupancy at the regulatory regions of genes that are required for these processes.

### Overexpression of SNF5 disrupts epigenetic regulation and enhances differentiation

**Figure 4:**
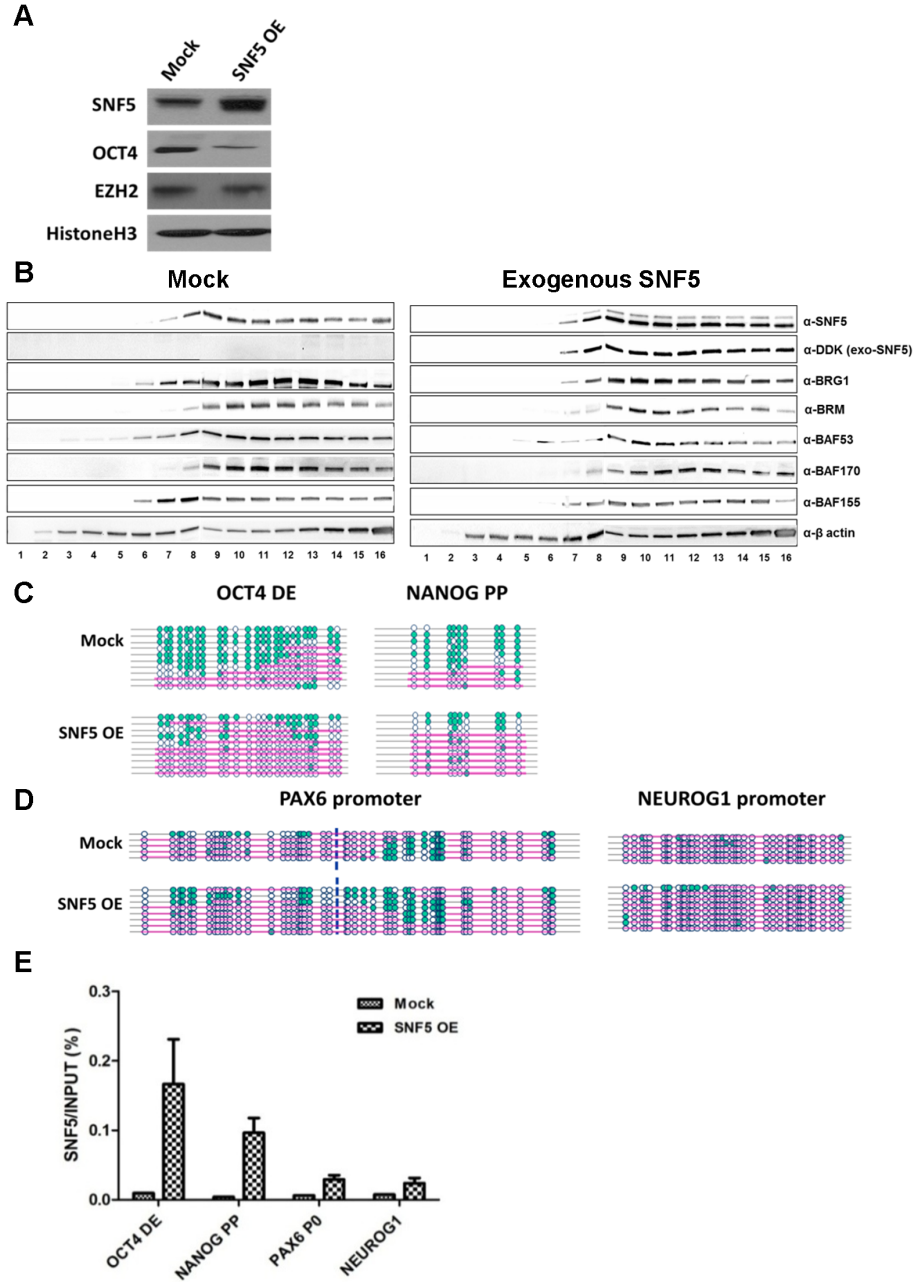
Overexpression of SNF5 disturbs epigenetic regulation and enhances differentiation. (A–D) Exogenous SNF5 was overexpressed in NCCIT cells and 72 h later, SNF5, OCT4, EZH2, and loading control histone H3 were analyzed by western blot (A). After exogenous SNF5 transfection, glycerol density centrifugation assay was performed. Fractions of 0.5 ml of the 10 ml 10∼30% glycerol gradient were collected and subjected to western bolt analysis for various BAF complex subunits (B). NOMe-seq was performed indicated OCT4 target regions (C and D). The data is representative of three biological experiments. (E) After overexpression, SNF5 binding at the DNA regulatory regions of OCT4 target genes were analyzed by quantitative PCR.

SNF5 overexpression in pluripotent cells resulted in the downregulation of OCT4 and NANOG whilst PAX6, NEUROG1 and EZH2 levels remained unchanged (Figure 4A and Figure S4A). To determine whether overexpressed exogenous SNF5 is part of a complex, we performed a glycerol gradient experiment after transfection with tagged exogenous SNF5. We found that exogenous SNF5 is recruited as a part of the BAF complex along with other subunits such as BRG1 and BRM (Figure 4B), suggesting that exogenous SNF5 forms a part of the complex and contributes to subsequent changes. We next performed NOMe-seq to determine nucleosome occupancy changes at DNA regulatory regions of OCT4 target genes after overexpression of SNF5 (Figure 4C and 4D). Although the *OCT4* DE and *NANOG* PP showed a decrease of NDRs from 47% to 21% and 71% to 20% respectively (Figure 4C), the *PAX6* and *NEUROG1* promoters displayed a minor expansion of NDRs (Figure 4D). We also found an increase in the binding of SNF5 and the other components at these loci, as a result of SNF5 overexpression (Figure 4E and Figure S4B), suggesting that these nucleosome occupancy changes are likely driven by SNF5 containing complexes. To further test this hypothesis, we overexpressed BRG1. Similar to the effect of SNF5 overexpression, an increase in BRG1 levels decreased OCT4 and NANOG expression and showed nucleosome occupancy at the *OCT4* DE and the *NANOG* PP (Figure S4C and Figure S4D). These data provide additional support for the concept that SNF5, as part of the BAF complex, plays a dual role in the regulation of nucleosome occupancy at OCT4 target genes by inserting nucleosomes at OCT4 activated genes and removing them at OCT4 repressed genes.

**Figure 5:**
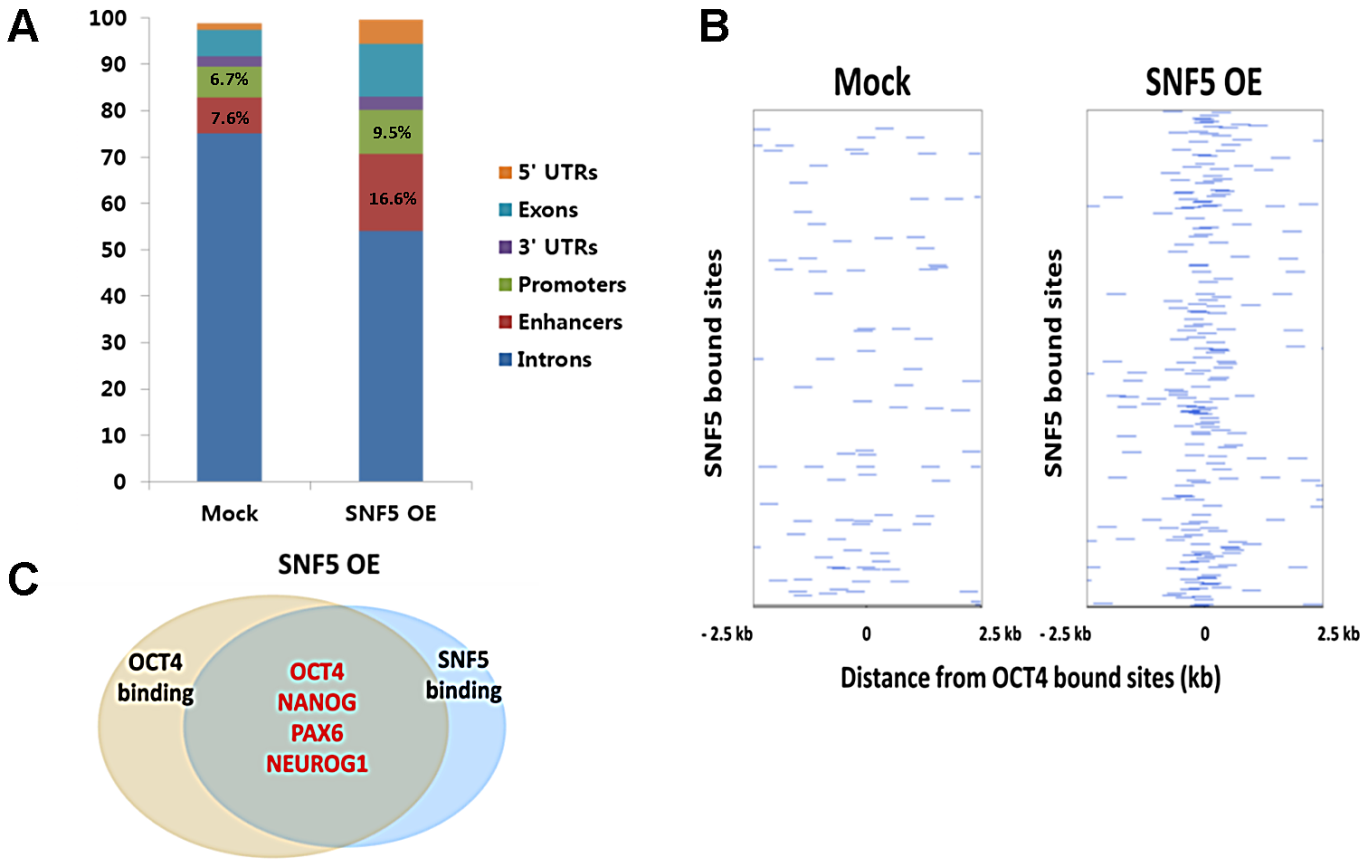
Overexpression of SNF5 alters SNF5 binding distribution, especially to OCT4 target genes. (A) Percentage distribution of ChIP-seq binding regions for SNF5 in control and overexpression state. (B) The binding plots show the localization of SNF5 bound sites relative to OCT4 bound sites. SNF5 bound sites (y axis) are displayed within a 5 kb window centered on the OCT4 bound site. Intensity at position 0 indicates that site overlap. (C) Venn diagram showing overlapping of OCT4 and SNF5 (the number of OCT4 only binding genes; 3412, the number of SNF5 only binding genes; 7185, and the number of both binding genes; 1862) bound genes after overexpression of SNF5 based on ChIP-seq data in NCCIT cells.

SNF5 ChIP-sequencing (ChIP-seq) was performed on NCCIT control cells and SNF5- overexpressing cells to assess the impact of SNF5 overexpression genome-wide. We found that >98% of SNF5 binding sites in the control and in overexpressing cells lay in regions containing gene promoters (defined as 2 kb regions centered at the TSS), enhancers, 5′UTRs, 3′UTRs, exons and introns (from hg19 RefSeq) (Figure 5A). Enhancers were identified as H3K4me1 peaks with two constraints, the distance between an enhancer and the nearest TSS should be less than 100 kb, and there is no overlap with any defined promoters in H1 cells. Notably, SNF5 overexpression altered SNF5 binding distribution and increased the enrichment at promoters and enhancers (Figure 5A). We also found that SNF5 binding at OCT4 targets significantly increased upon SNF5 overexpression; the average of normalized SNF5 ChIP reads showed 2.72 fold increase from 2.32 (mock) to 6.30 (overexpression). This suggests that alterations in SNF5 levels have direct effects on the regulation of OCT4 target genes due to its increased binding at DNA regulatory regions.

We defined SNF5 targets as those that are bound by SNF5 in any situation and have changed expression upon loss or gain of SNF5 (Table S1). By comparing SNF5 and OCT4 targets [51], we categorized genes into three groups: SNF5 exclusive targets (2,286 genes), OCT4 exclusive targets (921 genes) and shared SNF5 and OCT4 targets (332 genes). We found a relatively limited overlap between SNF5 and OCT4 binding sites (12.7%); this group contained a key set of OCT4 targets including core stemness genes such as *OCT4, NANOG*, and *SOX2*, and the master regulators of lineages *CDX2, PAX6, TBX3* and *ONECUT1*. Gene Ontology (GO) enrichment analysis for the three groups showed that the group containing targets shared by SNF5 and OCT4 included genes involved in the negative regulation of transcription and in cellular differentiation related pathways (Table S2). In an OCT4 centered binding plot, it is evident that OCT4 and SNF5 binding sites do not display significant overlap in control cells, while SNF5 overexpression seems to direct SNF5 to OCT4 target genes, resulting in greater overlap (Figure 5B). These binding patterns were more apparent in the SNF5 centered binding plot (Figure S5A). A dramatic 68% of all OCT4 target genes overlapped with SNF5 bound genes in cells overexpressing SNF5 (p-value 2.20×10^−75^), a group that includes crucial determinants of cell fate such as *OCT4, NANOG, PAX6* and *NEUROG1* (Figure 5C). The results presented thus far indicate that SNF5 binds a set of key OCT4 target genes when OCT4 is down-regulated upon RA induced differentiation or SNF5 overexpression. Although it is unclear whether SNF5 and OCT4 play antagonistic roles at the regulatory regions of these targets, it is apparent that the balance between the two controls important cellular functions.

To further examine whether there is an interesting human-mouse difference in BAF complex behavior, we carefully compared our human SNF5 ChIP-seq data and mouse Brg1 ChIP-seq in mouse ES cells [44]. In the mouse Brg1 ChIP-seq dataset, there were 10559 Brg1 binding sites identified, of which 4799 overlapped with promoters (NCBI GSE14344). In our SNF5 ChIP-seq data, we identified 26730 SNF5 binding sites, of which 9047 overlapped with human gene promoters. To assess whether SNF5 bound genes and Brg1 bound genes have significant overlaps, we first counted the number of genes common between human and mouse, which served as a background gene number for cross-species comparisons. There were 15578 genes that were common to human and mouse; of those 15578 genes, 7351 genes were bound by SNF5 (47%), 4001 genes were bound of Brg1 (26%), and 2400 genes were bounded by both SNF5 and Brg1 (15%) with an odds ratio = 2.0, and p-value<2.2xe-16 by chi-square test (Figure S5B). This finding could support the differences in the two studies: while Ho et al find that Brg1 knockdown results in reduced self-renewal of ES cells, we identify SNF5 knockdown as making cells less permissive to differentiation cues.

Further, we compared SNF5 bound genes with SUZ12 bound genes. Several studies have implicated polycomb repressive complexes in the maintenance of pluripotency. However, unexpectedly Suz12 showed a low degree of co-binding with Brg1 (30%) in the mouse system [44]. Human SUZ12 binding sites (27880) were downloaded from UCSC and these binding sites were determined to overlap with 14105 gene promoters. Surprisingly, 74% of SNF5 bound genes were co-bound by SUZ12 (odds ratio = 2.71, and p-value<2.2xe-16) (Figure S5C). This provides further evidence for differences in BAF complex behavior in the human and mouse systems. The discrepancy between human and mouse might be related to functional evolutionary diversification of these complexes and a deep understanding remains for future study.

### SNF5 controls the balance between pluripotency and differentiation

**Figure 6:**
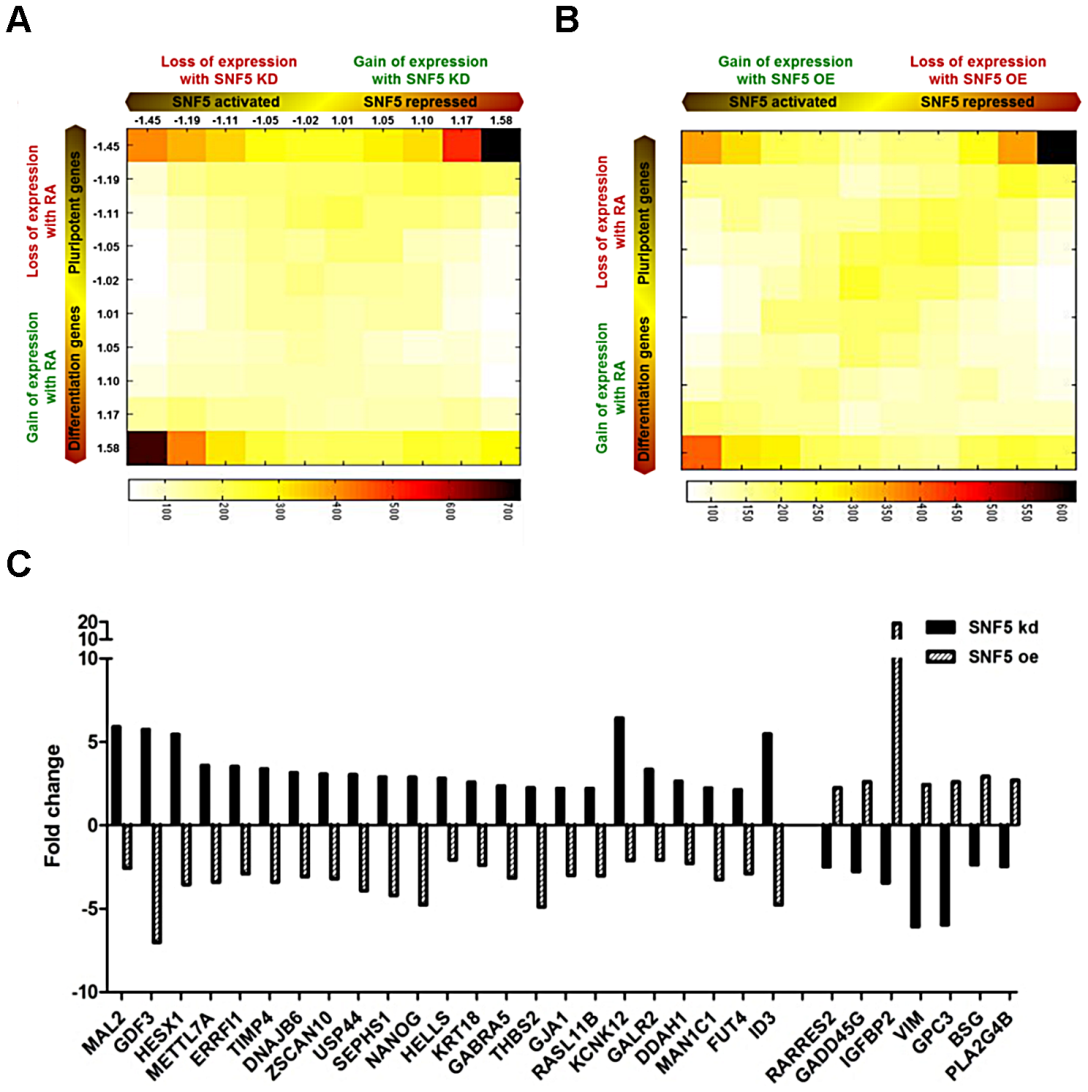
SNF5 controls the balance between pluripotency and differentiation. (A and B) 2D matrix and heat plots depicting gene expression changes in SNF5 knockdown/SNF5 overexpression and RA 7 d treated NCCIT cells. Axes indicate degree of fold change, from the middle of axis. The numbers indicate the median fold change of genes in each column or row. The intensity of each square represents the number of genes that fall in that square. (C) Fold change of SNF5 target genes (over two fold changes in opposite direction) among previously defined ES signature genes [51].

We performed and analyzed a total of 12 gene expression microarrays including duplicates of RA treated samples and samples from cells in which SNF5 was knocked down or overexpressed to obtain a comprehensive understanding of the role of SNF5 in pluripotency and differentiation. We identified statistically significant gene expression changes (false discovery rate<0.05) that occur due to the changes in SNF5 levels and RA treatment and performed GO analysis (Table S3, S4 and S5) using the gene expression data (Figure S6A, S6B and S6C). The data are presented as a two-dimensional (2D) gene density heat map [52] of the genes regulated by SNF5 and RA (Figure 6A and 6B). The results showed that SNF5 and RA treatment both activate (lower left corner) and repress (upper right corner) the same set of genes (Figure 6A and 6B). SNF5 had a distinct dual role in controlling pluripotency-associated genes (Figure 6A and 6B upper corners), whereas it mainly activated differentiation-related genes (Figure 6A and 6B lower left corners). In agreement with our previous results, the upper right corner included well-known pluripotency genes, such as *NANOG*, *ZNF42, DPPA3, DPPA4* and *GDF3* in both the SNF5 knockdown and overexpression heat maps (Figure 6A and 6B). We observed that few genes are shared in common between the groups that are affected by RA treatment and SNF5 knockdown (lower right corner in Figure 6A). This could be explained as RA treatment and SNF5 knockdown cause opposite effects on cell differentiation and, hence, on the expression of stemness gene. We also looked at genes that are co-expressed on RA treatment and SNF5 overexpression (lower left corner in Figure 6B). The number of genes co-regulated is much larger as seen by the color density and these include genes that are important in multicellular organismal development (Table S6).

Further, several genes that showed over two-fold changes in expression levels belonged to a previously defined ES signature [51] (Figure 6C). Several interesting genes such as the transcriptional repressor *HESX1*, a negative regulator of EGFR signaling *ERRFI1* and the lymphoid-specific helicase *HELLS* were repressed by SNF5. *HELLS* is known to play an essential role in development and survival and is required for *de novo* and maintenance DNA methylation [53]. Among the SNF5 activated genes, *GADD45G* is known to be a key regulator of cell growth and apoptosis, and *IGFBP2* has been established as an inhibitor of IGF-mediated growth and developmental rates. Since SNF5 overexpression itself forced premature differentiation (Figure 4) and SNF5 knockdown blocked differentiation (Figure 3D and 3E) and reduced cell survival under differentiation signal (Figure 3F), we strongly believe that there is a specific association between SNF5 and differentiation rather than a general requirement for chromatin modifications during differentiation. Taken together, these data demonstrate that SNF5 plays a balancing role in regulating OCT4 levels in the pluripotent state.

SNF5 is essential for cell differentiation and acts by repressing “stemness” gene networks and by activating the networks involved in differentiation, consequently allowing for cell survival during the differentiation process.

## Discussion

**Figure 7:**
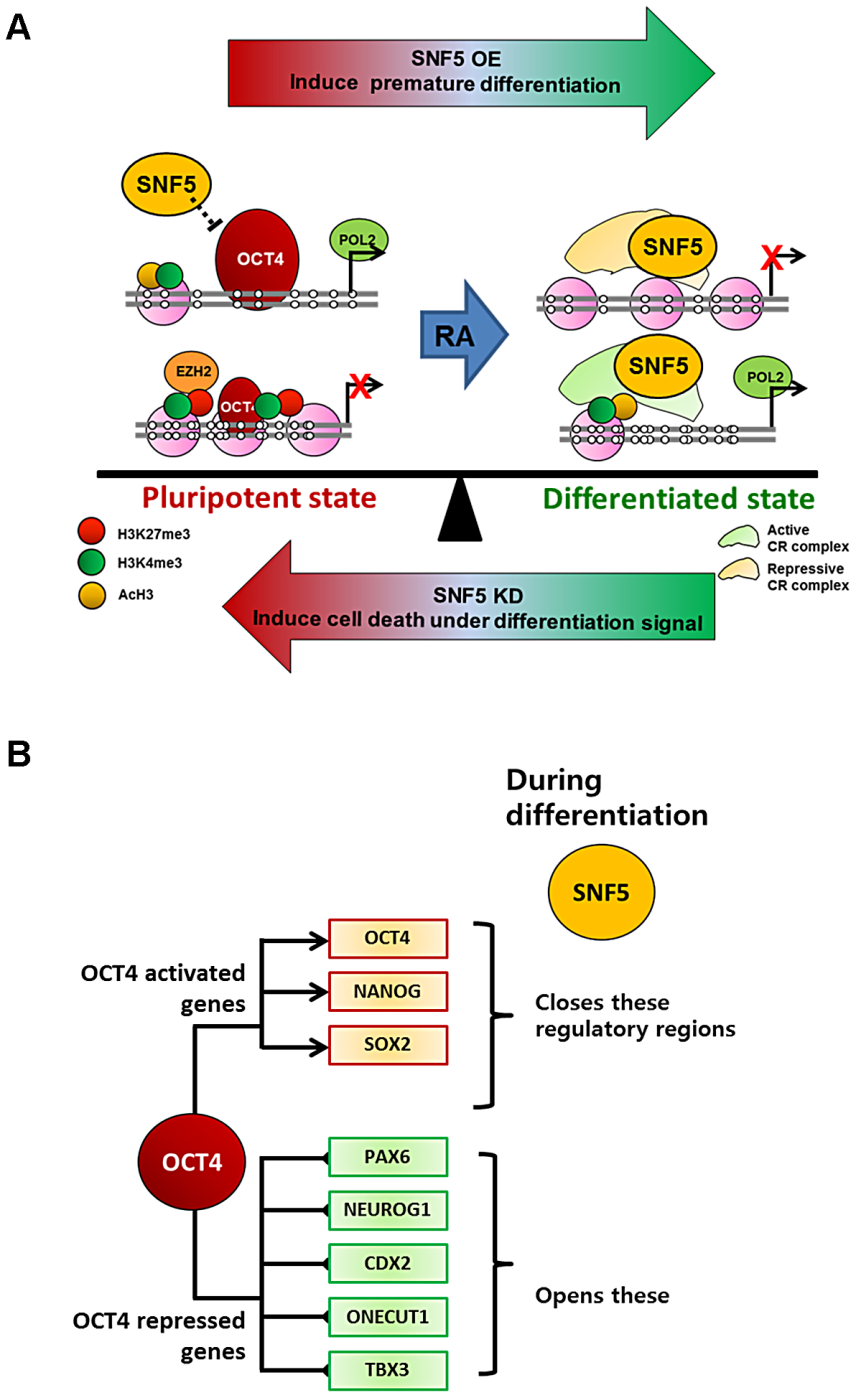
SNF5 is a key executor of epigenetic regulation in pluripotency and differentiation. (A and B) Differentiation signals cause recruitment of SNF5 to both OCT4 activated and repressed target genes with distinctive roles (closing or generating NDRs) dependent on cellular context. Changes in exogenous SNF5 levels disrupt the balance between pluripotent and differentiated states. (Refer to Discussion for a detailed explanation).

In this study, we demonstrate that SNF5 plays a critical role in controlling the delicate balance between pluripotency and differentiation by altering nucleosome occupancy at OCT4 target genes (summarized in Figure 7A and 7B). In the pluripotent state, the DNA regulatory regions of OCT4 active targets (*OCT4* DE and *NANOG* PP) have an open chromatin structure, while the OCT4 repressive targets (*PAX6* and *NEUROG1* promoters) display a closed configuration, with OCT4 occupancy and appropriate histone modifications. When cells receive a differentiation signal such as RA, the NDRs at the OCT4 active genes collapse and *de novo* NDRs are established at the OCT4 repressive targets accompanied by the recruitment of SNF5. Changes in exogenous SNF5 levels disrupt the balance between pluripotent and differentiated states. SNF5 knockdown results in the upregulation of OCT4 levels and these cells fail to differentiate on RA stimulation but rather undergo massive cell death. In contrast, overexpression of SNF5 represses OCT4 levels and leads to premature differentiation. Our genome-wide study supports this interpretation since the chromatin remodeler SNF5 and the natural morphogen RA activate and repress essentially the same set of genes. Therefore, our results suggest that SNF5 is an essential executor of the balance between pluripotency and differentiation.

Antagonism between polycomb group (EZH2) and SWI/SNF (SNF5) has been described in certain contexts [32], [40], [41], [54], although the mechanism underlying such antagonism remains controversial and is likely to be complex [24]. For example, Ho et al [52] suggest that the mouse esBAF complex maintains the accessibility of Stat3 to LIF targets by preventing the application of the repressive polycomb marker H3K27me3, while at the same time this esBAF also shows synergy with other polycomb target such as the *Hox* genes. Herein, we do not observe direct antagonism between the SNF5 and EZH2 molecules themselves (Figure 3A, 3D, Figure S3D and Figure 4A) and do not find any major directional change in the expression of the module defined by genes marked by H3K27me3, a histone mark characteristic of polycomb repression (Figure S6D and S6E). Thus, the detailed molecular mechanism underlying the relationship between SWI/SNF and polycomb complexes in pluripotent cells requires further investigation.

Notably 60.0% and 52.1% of differentially expressed genes upon SNF5 knockdown and overexpression, respectively, were directly bound by SNF5, suggesting that the majority of phenotypic changes that we observe are directly related to the action of SNF5. We further showed that SNF5 is essential for cell survival during differentiation, as evidenced by two experiments (knocking down SNF5 and then inducing differentiation and knocking down SNF5 after the initiation of differentiation), which is also supported by a previous report that showed homozygous knockout of SNF5 in a mouse results in embryonic lethality [55].

Still, the mechanisms by which SNF5 changes affinities for its target genes and partners with transcription factors and exogenous cellular signals remain unknown. Aberrant loss or gain of SNF5 expression could result in new combinatorial assemblies of SWI/SNF, thereby potentially impacting several molecular and phenotypic changes. SNF5 has primarily been shown to be dedicated to the BAF complex and to enhance its remodeling activity [56]. Our results also show that SNF5 localization to OCT4 target sites correlates with that of other BAF subunits. Although, glycerol gradient experiment supports that this exogenous SNF5 is recruited as a part of the BAF complex along with other subunits such as BRG1 and BRM (Figure 4B), we cannot exclude the possibility that SNF5 may have functions independent of BAF complexes. In this regard, a study showed that SNF5 is dispensable for BAF assembly in some cancer cells [57] and another study showed that SWI/SNF facilitates gene regulation through a greater diversity of interactions [58]. More studies are required to elucidate this issue.

The SWI/SNF complex is well known for its importance in polymerases binding during transcriptional activation and has been proposed as a part of the POL2 complex [59], [60], [61], [62]. Regardless, its repressive role has been overlooked in the chromatin remodeling field. Our study highlights that SNF5 acts as a dual agent for the fine-tuning of its target genes' expression during differentiation. These results provide another insightful view of the chromatin remodeler's diverse functions.

It is well known that genome-wide changes in chromatin structure occur during differentiation and a number of chromatin remodelers have been associated with the pluripotency state. However, little is known about the function of chromatin remodelers during the actual process of differentiation and their interactions with transcription factors. Our results show, for the first time, that the chromatin remodeler SNF5 plays a crucial role in the regulation of nucleosome occupancy at OCT4 target genes and that it is required for the switch between pluripotency and differentiation. Gaining a further understanding of chromatin remodeling complexes could allow us to determine the mechanism by which genetic mutations in this class of enzymes is implicated in diseases such as cancer. Collectively our results have clear implications for stem cell and cancer biology and may provide novel insights for regenerative therapies.

## Materials and Methods

### Accession numbers

Microrarray data: GSE35909, ChIP-seq data: GSE36134

### Nucleosome occupancy and methylome sequencing (NOMe-seq)

Cells were trypsinized and cell pellets were washed in PBS and resuspended in 1 mL ice-cold Nuclei Buffer (10 mM Tris, pH 7.4, 10 mM NaCl, 3 mM MgCl2, 0.1 mM EDTA and 0.5% NP40, plus protease inhibitors) per 5×10^6^ cells. Nuclei were recovered by centrifugation, washed in Nuclei Wash Buffer (10 mM Tris, pH 7.4, 10 mM NaCl, 3 mM MgCl2, 0.1 mM EDTA containing protease inhibitors) and reseuspended at a concentration of 4.0×10^6^ cells/mL in 1X M.CviPI reaction buffers. Purified genomic DNA were treated with 200 U of M.CviPI for 15 min at 37°C. Reactions were stopped by the addition of an equal volume of Stop Solution (20 nM Tris HCl, pH 7.9, 600 mM NaCl, 1%SDS, 10 mM EDTA, 400 ug/ml Proteinase K) and incubated at 55°C overnight. DNA was purified by phenol/chloroform extraction and ethanol precipitation. Bisulfite conversion was performed suing the Epitect Bisulfite Kit (Qiagen). Molecules were cloned using the Topo TA Kit (Invitrogen), both according to the manufacturers' instructions.

### RNA extraction and reverse transcription PCR

Total RNA was extracted using Trizol reagent, digested with DNAseI, and reverse transcribed with SuperScript III Reverse Transcriptase (Invitrogen). Amplification of cDNA was performed on the an Opticon light real-time PCR cycler (BioRad) using KAPA Probe Fast qPCR Mix (Kapa Biosystems) using recommended conditions. OCT4 5′-CCCTGGTGCCGTGAAGC, 3′-TTGCTCGAGTTCTTTCTGCAGA, Probe-AGCAAAACCCGGAGGAGTCCCAGG, NANOG RT 5′-GCAGAAGGCCTCAGCACCTA, 3′ -AGTCGGGTTCACCAGGCAT, Probe-CTACCCCAGCCTTTACTCTTCCTACCACCA, PAX6 RT 5′- CCTATGCCCAGCTTCACCAT, 3′- GGCAGCATGCAGGAGTATGAG, NEUROG1 RT 5′- GCAGTGACCTATCCGGCTTC, 3′- GGAGGCTGCCTGTTGGAGT

### Western blot analysis

Cell lysates were boiled in Laemmli sample buffer for 3 min, and 30 µg of each protein were subjected to SDS-PAGE. Antibodies against SNF5 (ab12167), BRG1 (ab4081), BRM (ab15597), and BAF53b (ab103771) were purchased from Abcam, Inc (Cambridge, MA). Antibodies against BAF155 (sc-10756) and BAF170 (sc-10757) were purchased from Santa Cruz Biotechnology (Santa Cruz, CA). Antibodies against beta-actin (a2228) were purchased from Sigma-Aldrich, Inc (St. Louis, MO), while antibodies against DDK fusion tag (TA50011) were purchased from OriGene technologies, Inc (Rockville, MD).

### Chromatin immunoprecipitation assay

Chromatin immunoprecipitation assays were done according to the Upstate Biotechnology instructions. For each ChIP, 100 ug DNA sheared by a sonicator was precleared with salmon-sperm DNA-saturated protein A sepharose, and then precipitated by histone H3 antibody and others. After IP, recovered chromatin fragments were subjected real-time PCR. IgG control experiments are performed for all ChIPs and accounted for in the IP/Input by presenting the results as (IP- IgG)/(Input-IgG). OCT4 DE 5′- GAGGATGGCAAGCTGAGAAA, 3′ – CTCAATCCCCAGGACAGAAC, NANOG PP 5′ – TTGTTGCTGGGTTTGTCTTCAG, 3′ – AAAGTAGCTGCAGAGTAACCCAGACT, PAX6 P0 5′- TGGAGTTGGCAAGAAAGGAC, 3′- GAGCGGTCAAGTGAAGGTTT, PAX6 P1 5′- TGTTGCGGAGTGATTAGTGG, 3′- TTGGTGATGGCTCAAGTGTG, NEUROG1 P 5′- CGGTAATTACGGGCACACTC, 3′- CTTAAGTACCCGGCGCAAC.

### Small interfering RNA transfection

Cells were transfected with scrambled or target gene-specific small interfering RNA (siRNA) using Lipofectamine LTX (Invitrogen). siRNAs specifically targeting SNF5 and OCT4 were purchased from Dharmacon.

### shRNA infection

The constructs of shSNF5 were purchased from Open Biosystems. For lentivirus production, the vesicular stomatitis virus envelope protein G expression construct pMD.G1, the packaging vector pCMVΔR8.91 and the transfer vector pLJM1 were used. Infected NCCIT derivative cells, stably expressing shSNF5, were selected in the presence of 1.25 µg/ml puromycin.

### Apoptosis assay

Cellular apoptosis was measured by Annexin-V and Propidium Iodide (PI) staining using Annexin V-FITC Apoptosis Detection Kit (MBL), according to the manufacturer's protocol.

### Ectopic gene expression

Cells were transfected with mock or exogenous SNF5 expression vectors using Lipofectamine LTX (Invitrogen). Exogenous SNF5 expression vectors were purchased from Origene.

### Preparation of NCCIT nuclear proteins

NCCIT cells with or without SNF5 overexpression were grown to confluence and lysed in Buffer A (10 mM Tris-Cl pH7.4, 10 mM NaCl, 3 mM MgCl2, 0.1 mM EDTA, 0.5% NP-40, 1 mM dithiothreitol and protease inhibitors (Roche)) on ice. Nuclei were sedimented by centrifugation (1000×g for 5 min) and washed with ice cold Buffer B (10 mM HEPES pH7.6, 25 mM KCl, 0.1 mM EDTA, 1 mM dithiothreitol and 10% glycerol). Nuclei were then resuspended in Buffer C (10 mM HEPES pH7.6, 3 mM MgCl2, 100 mM KCl, 0.1 mM EDTA, 1 mM dithiothreitol, 10% glycerol and protease inhibitors) and lysed by the addition of ammonium sulfate to a final concentration of 0.3 M. After ultracentrifugation (100,000×g for 20 min), soluble nuclear proteins in the supernatant were collected and precipitated with 0.3 g/ml ammonium sulfate for 20 min on ice. Protein precipitate was pelleted by centrifugation (15,000×g for 20 min) and dissolved in HEMG-0 buffer (25 mM HEPES pH7.9, 0.1 mM EDTA, 12.5 mM MgCl2, 100 mM KCl, 1 mM dithiothreitol and protease inhibitors).

### Glycerol gradient analysis

This experiment was performed as described by Lessard J et at. [63] with minor modifications. Briefly, 800 µg of nuclear proteins prepared as described above were fractionated through 10 ml glycerol density gradient solution (10 to 30% glycerol, 25 mM HEPES pH7.9, 0.1 mM EDTA, 12.5 mM MgCl2, 100 mM KCl, and 1 mM dithiothreitol) by ultracentrifugation (40,000 RPM, 16 hours) at 4°C using a SW-41 swing bucket rotor (Beckman). Sixteen fractions with equal volume were then harvested starting from the top to the bottom of the centrifuge tube. Proteins from each fraction were concentrated by trichloroacetic acid precipitation, dissolved in 1X SDS-PAGE loading buffer (50 mM Tris-Cl pH6.8, 2% sodium dodecyl sulfate, 0.1% bromophenol blue, 10% glycerol and 100 mM dithiothreitol) and resolved on 4 to 15% gradient SDS-PAGE gels (Bio-Rad) before Western blot analysis.

### ChIP sequencing

ChIP-seq samples were generated using 1×10^8^ cells using an antibody directed toward SNF5 and OCT4 ChIPed DNA (20 ng) was used to generate libraries using previously described methods [64]. Amplicons used for sequencing on an Illumina GA II machine. Reads were mapped using the mapq software. Robustly annotated transcription start sites were taken from the UCSC Known Genes resource. We compared the distribution of SNF5 binding sites between SNF5 in the mock sample and in the SNF5 over-expression sample. We applied MACS (Yong Zhang et al. Genome Biology, 2008) to identify SNF5 binding sites in SNF5 mock and over-expression states. The number of SNF5 binding sites was 11,411 in the mock and 26,730 in the SNF5 over-expression state (p-value<10e-4).

### Genome-wide gene expression array

Purified RNA was processed and hybridized onto Illumina Human HT-12 v4 array according to the manufacturer's instructions. We applied the lumi package [65], specially designed for the Illumina array analysis, to the obtained data for variance stabilizing transformation. We then performed the quantile normalization across the arrays, and applied the Limma package [66] to identify differentially expressed genes in each of the experimental group pairs, which are NCCIT+Consh VS NCCIT+SNF5sh, NCCIT+Mock VS NCCIT+SNF5oe and NCCIT+Mock VS NCCIT+ RA 7 d. For the differential analysis for SNF5 over-expression and knockdown respectively, we carried out quantile normalization by R package lumi [65] to make gene expression between different Illumina arrays comparable, and then used R package limma [67] to identify differentially expressed genes with adjusted p-value<0.01.

### Generation of 2D matrices and average module gene expression

We performed these analysis as described previously [68].

### Correlation between DNA methylation, DNAseI hypersensitivity, and OCT4 binding

DNA methylation and DNAseI hypersensitivity data for H1 embryonic stem cells was obtained from the ENCODE and GEO data. The DNAseI hypersensitive peaks were identified as signal peaks within FDR 0.5% hypersensitive zones. The OCT4 peaks in H1 embryonic stem cells were obtained from GEO (GSM518373). For the DNA methylation data, we filtered out the regions with a bed score less than 1000, in order to maintain only DNA methylated regions. Next we counted all the DNA methylated genomic regions that have any overlap with the DNAseI hypersensitive peaks and with OCT4 binding peaks to generate the Venn diagram.

## Supporting Information

Figure S1 Scheme of primer sets for NOMe-seq and ChIP assays. The red asterisk indicates OCT4 binding site based on references and published OCT4 ChIP-seq data [69] (GSM518373) (A). Pluripotent human embryonic stem cell H1 and carcinoma NCCIT cells were exposed to 10 uM of retinoic acid (RA) for the indicated days. The endogenous DNA methylation level of PAX6, and NEUROG1 promoters was determined by NOMe-seq assay. White circles represent unmethylated, black circles represent methylated CpG sites (B). The nucleosome occupancy of SOX2, CDX2, TBX3 and ONECUT1 were determined by NOMe-seq (F). PAX6 and NEUROG1 nucleosome occupancy were studied in H9, H9 and glioblastoma 248 cells (C and D). The expression levels of NEUROG1, SOX2, CDX2, TBX3 and ONECUT1 were determined by quantitative PCR (normalized to PCNA) at each indicated time point and cell lines using specific primers and probes (E and G). Quantitative PCR data were combined of three biological experiments (the mean +SEM).
(PDF)

Figure S2 Chromatin from NCCIT cells was immunoprecipitated with anti-H3K27me3 (A), anti-H3K4me3 (B), anti- AcH3 (C), anti- H3 (D), anti-Pol2 (E), anti-SNF5 (F), and anti-BAF170 (G) antibodies and their binding at the DNA regulatory regions of OCT4 target genes were analyzed by quantitative PCR. Quantitative PCR data were combined of three biological experiments (the mean +SEM).
(PDF)

Figure S3 72 h post-transfection with SNF5 siRNA, OCT4 siRNA and BRG1 siRNA in NCCIT cells, mRNA levels of OCT4, NANOG, PAX6 and NEUROG1 and protein of OCT4, NANOG, BRG1 and histone H2 were analyzed by quantitative PCR (A) and western blot (B). Quantitative PCR data were combined of three biological experiments. After transient knockdown of OCT4 and BRG1, NOMe-seq was performed for PAX6 and NEUROG1 promoters, OCT4 DE and NANOG PP (C). 21 d post-infection with SNF5 shRNA lentivirus in NCCIT cells, mRNA levels of SNF5, EZH2, OCT4, NANOG, PAX6 and NEUROG1 were analyzed by quantitative PCR (D). Quantitative PCR data were combined of three biological experiments. 7 d post-infection with SNF5 shRNA lentivirus in H1 cells, mRNA levels of OCT4, NANOG, PAX6 and NEUROG1 were analyzed by quantitative PCR (E).
(PDF)

Figure S4 72 h post-transfection with exogenous SNF5 overexpression vector in NCCIT cells, mRNA levels of OCT4, NANOG, PAX6 and NEUROG1 were analyzed by quantitative PCR (A). Quantitative PCR data were combined of two biological experiments. After SNF5 overexpression, BRG1 and BAF170 binding at the DNA regulatory regions of OCT4 target genes were analyzed by quantitative PCR (B). After BRG1 overexpression, OCT4 targets mRNA quantitative PCR (C) and NOMe-seq (D) performed.
(PDF)

Figure S5 The binding plots show the localization of OCT4 bound sites relative to SNF5 bound sites (A). OCT4 bound sites (y axis) are displayed within a 5 kb window centered on the SNF5 bound site. Intensity at position 0 indicates that site overlap. Mouse Brg1 binding sites were downloaded from NCBI GSE14344 and compared with our SNF5 ChIP-seq data (B). SUZ12 binding sites (wgEncodeBroadHistoneH1hescSuz12051317Pk.broadPeak.gz) were downloaded from UCSC and compared with our SNF5 ChIP-seq data (C). By chi-square test, SNF5 bound genes and Suz12 bound genes significantly overlap with each other, with odds ratio = 2.71, and p-value<2.2×e-16. The Venn Diagram represented that 6736 (74.4%) were also bound by SUZ12 out of 9047 SNF5 bound genes.
(PDF)

Figure S6 Scatter plots comparing global gene expression profiles between SNF5 shRNA #2 (shSNF5#2) and control shRNA (shControl) cell lines (A), between SNF5 overexpression (SNF5 OE) and Mock cell lines (B), and between RA treated and control NCCIT cells (C). Individual genes within the H3K27me3 bounded target gene module were analyzed in SNF5 knockdown (D) and overexpression (E) states.
(PDF)

Table S1 The SNF5 target gene list.
(XLSX)

Table S2 The GO analysis of SNF5 and OCT4 targets.
(XLSX)

Table S3 The GO analysis and gene list upon RA treatment.
(XLSX)

Table S4 The GO analysis of manipulation of SNF5 level by knockdown or overexpression.
(XLSX)

Table S5 The gene list of SNF5 level with RA treatment.
(XLSX)

Table S6 The GO analysis and gene list of 2D plot.
(XLSX)
