# Supplementary figures and images for: SNF5 Is an Essential Executor of Epigenetic Regulation during Differentiation
Source: PLoS Genet. 2013 Apr 25;9(4):e1003459. doi: 10.1371/journal.pgen.1003459 (PMC3636213; doi:10.1371/journal.pgen.1003459)

**A**

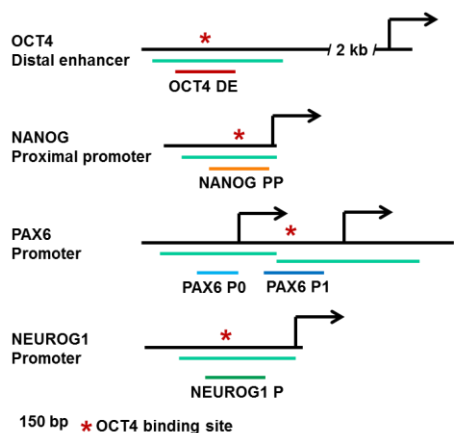

**B**

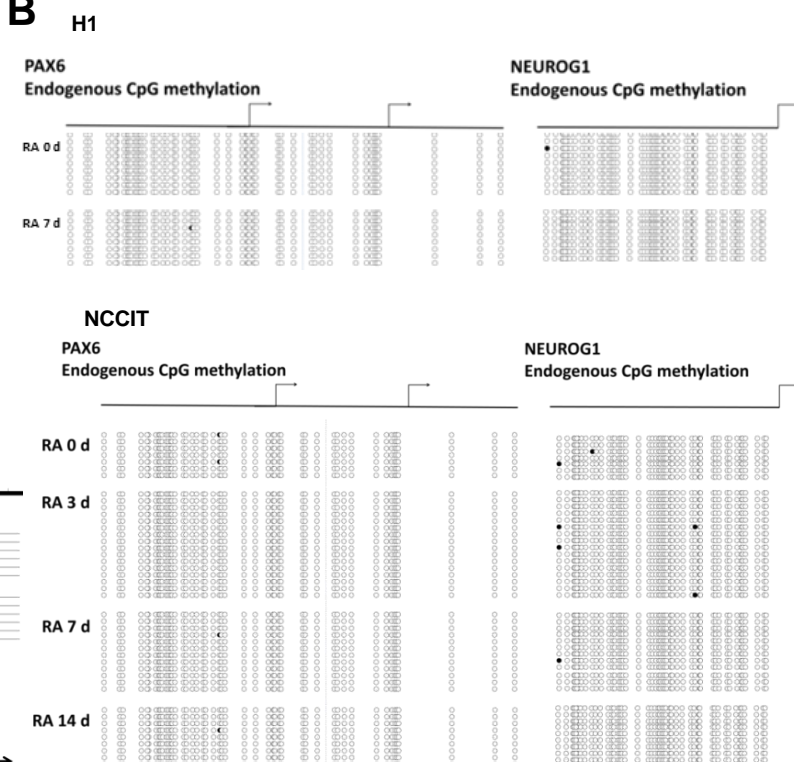

**C**

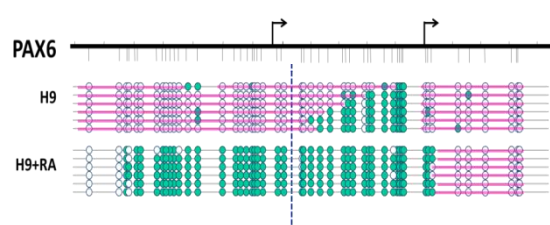

**D**

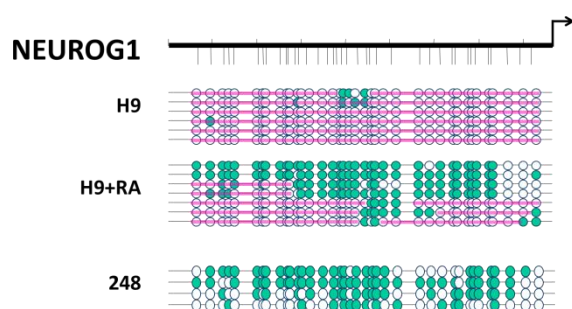

**F**

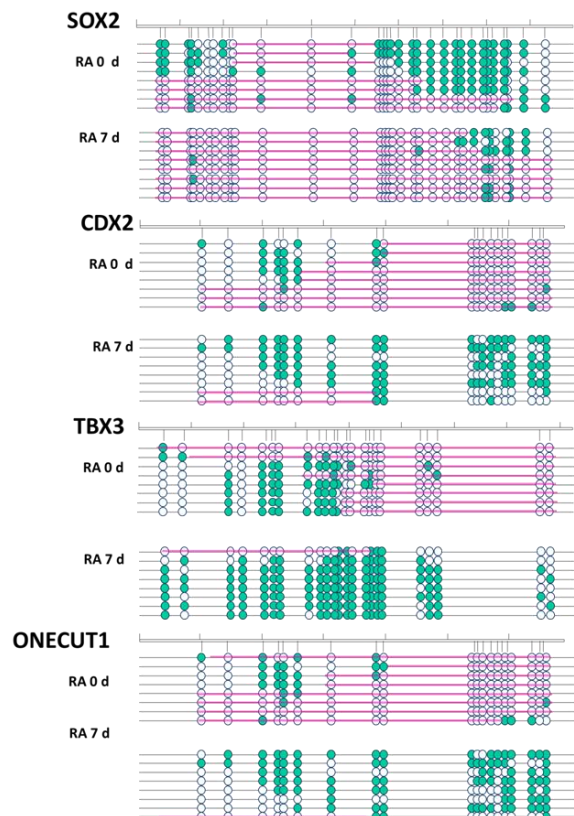

**E**

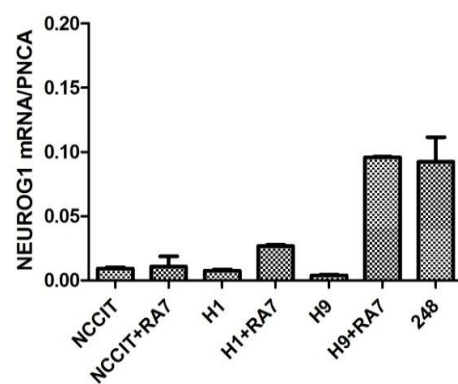

**G**

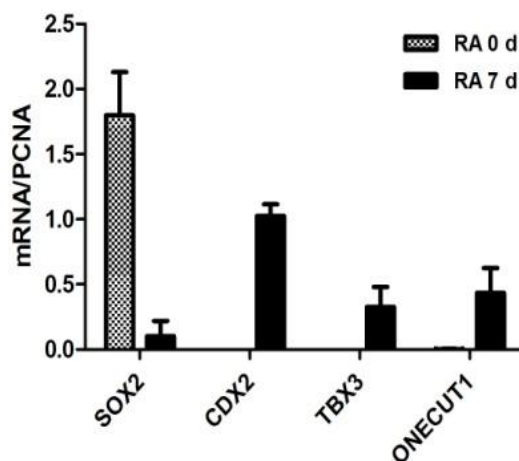

Supplement: Figure S1 — Scheme of primer sets for NOMe-seq and ChIP assays. The red asterisk indicates OCT4 binding site based on references and published OCT4 ChIP-seq data [69] (GSM518373) (A). Pluripotent human embryonic stem cell H1 and carcinoma NCCIT cells were exposed to 10 uM of retinoic acid (RA) for the indicated days. The endogenous DNA methylation level of PAX6, and NEUROG1 promoters was determined by NOMe-seq assay. White circles represent unmethylated, black circles represent methylated CpG sites (B). The nucleosome occupancy of SOX2, CDX2, TBX3 and ONECUT1 were determined by NOMe-seq (F). PAX6 and NEUROG1 nucleosome occupancy were studied in H9, H9 and glioblastoma 248 cells (C and D). The expression levels of NEUROG1, SOX2, CDX2, TBX3 and ONECUT1 were determined by quantitative PCR (normalized to PCNA) at each indicated time point and cell lines using specific primers and probes (E and G). Quantitative PCR data were combined of three biological experiments (the mean +SEM). (PDF) [file pgen.1003459.s001.pdf]

**A**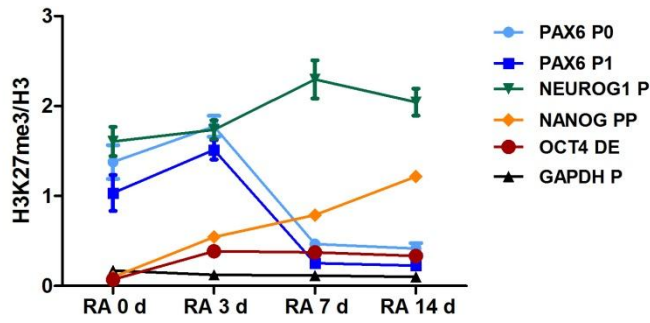**B**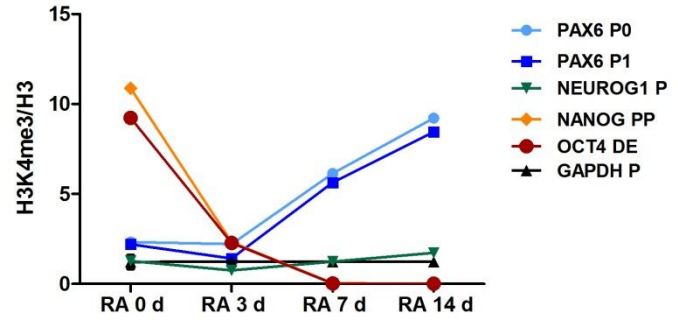**C**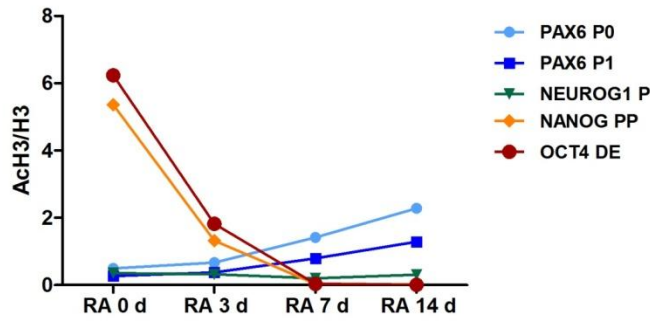**D**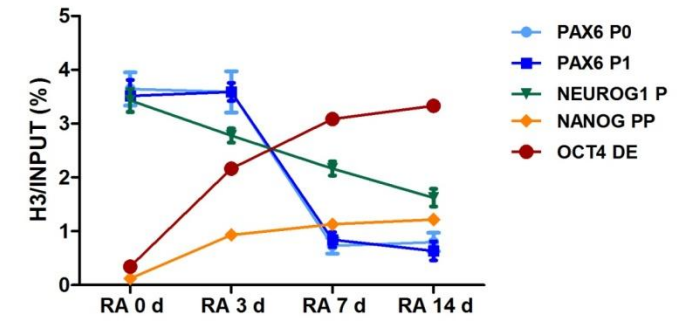**E**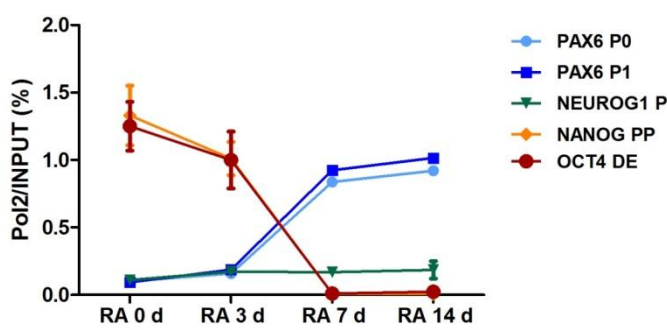**F**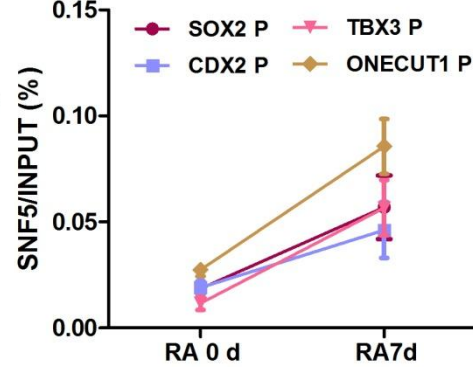**G**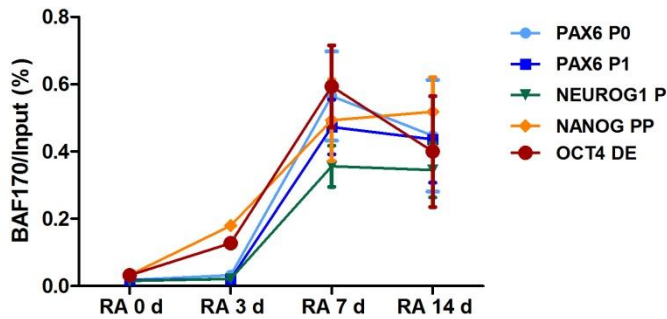

Supplement: Figure S2 — Chromatin from NCCIT cells was immunoprecipitated with anti-H3K27me3 (A), anti-H3K4me3 (B), anti- AcH3 (C), anti- H3 (D), anti-Pol2 (E), anti-SNF5 (F), and anti-BAF170 (G) antibodies and their binding at the DNA regulatory regions of OCT4 target genes were analyzed by quantitative PCR. Quantitative PCR data were combined of three biological experiments (the mean +SEM). (PDF) [file pgen.1003459.s002.pdf]

**A**

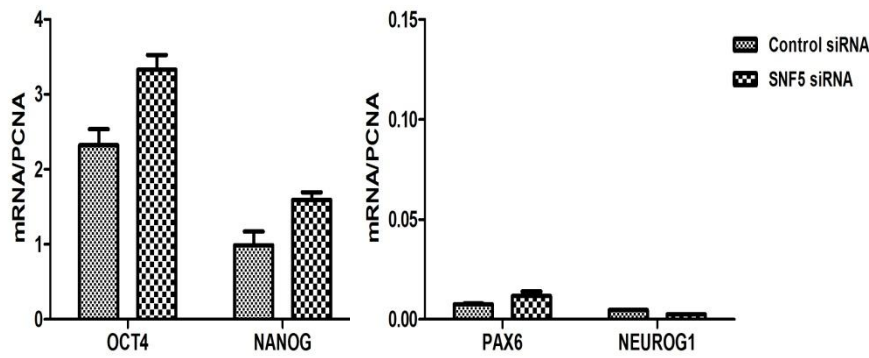

**B**

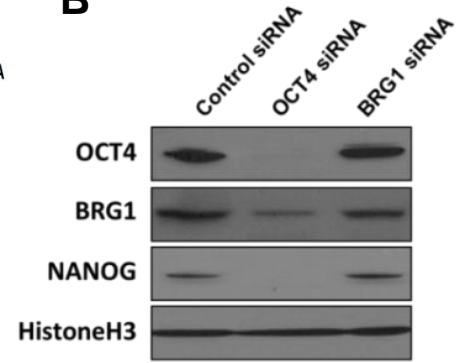

**C**

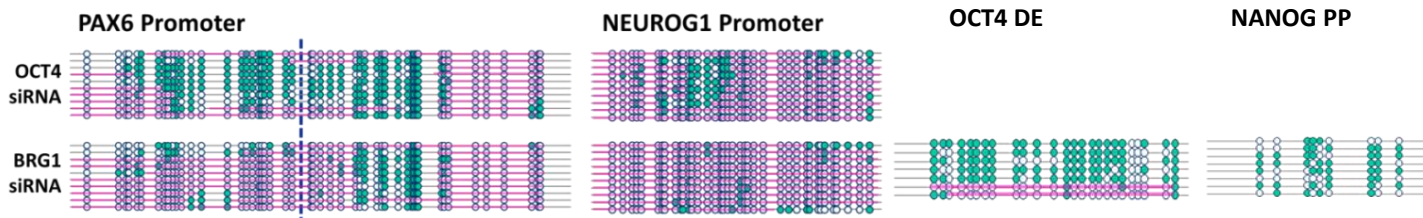

**D**

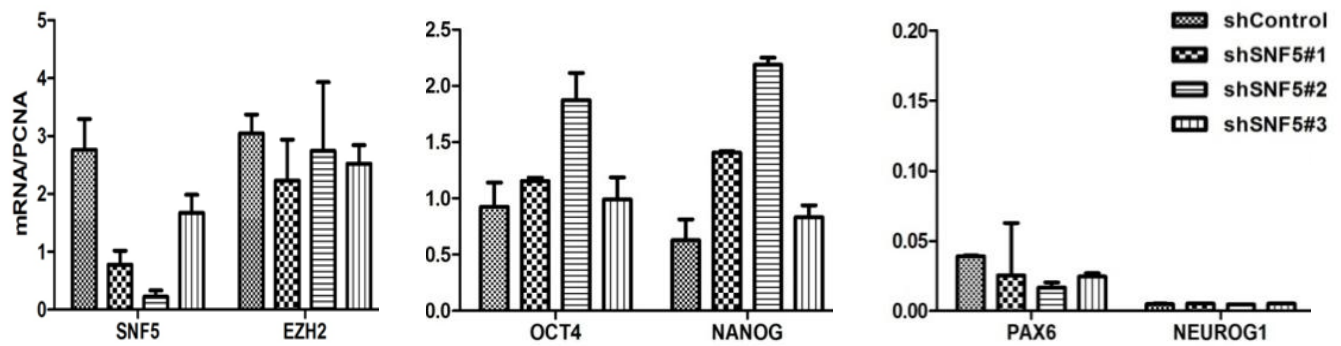

**E**

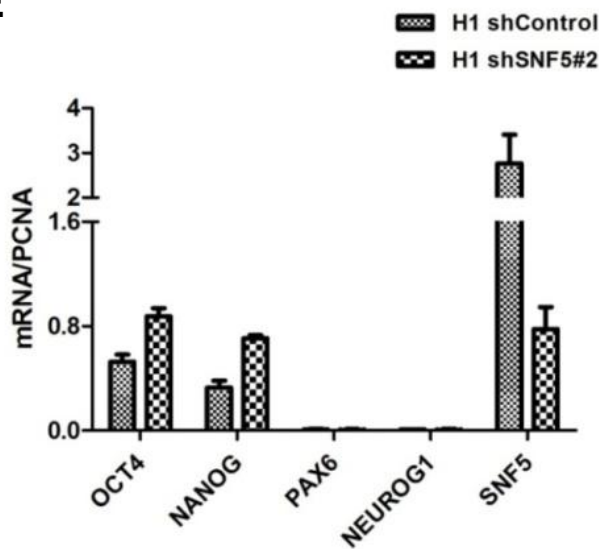

Supplement: Figure S3 — 72 h post-transfection with SNF5 siRNA, OCT4 siRNA and BRG1 siRNA in NCCIT cells, mRNA levels of OCT4, NANOG, PAX6 and NEUROG1 and protein of OCT4, NANOG, BRG1 and histone H2 were analyzed by quantitative PCR (A) and western blot (B). Quantitative PCR data were combined of three biological experiments. After transient knockdown of OCT4 and BRG1, NOMe-seq was performed for PAX6 and NEUROG1 promoters, OCT4 DE and NANOG PP (C). 21 d post-infection with SNF5 shRNA lentivirus in NCCIT cells, mRNA levels of SNF5, EZH2, OCT4, NANOG, PAX6 and NEUROG1 were analyzed by quantitative PCR (D). Quantitative PCR data were combined of three biological experiments. 7 d post-infection with SNF5 shRNA lentivirus in H1 cells, mRNA levels of OCT4, NANOG, PAX6 and NEUROG1 were analyzed by quantitative PCR (E). (PDF) [file pgen.1003459.s003.pdf]

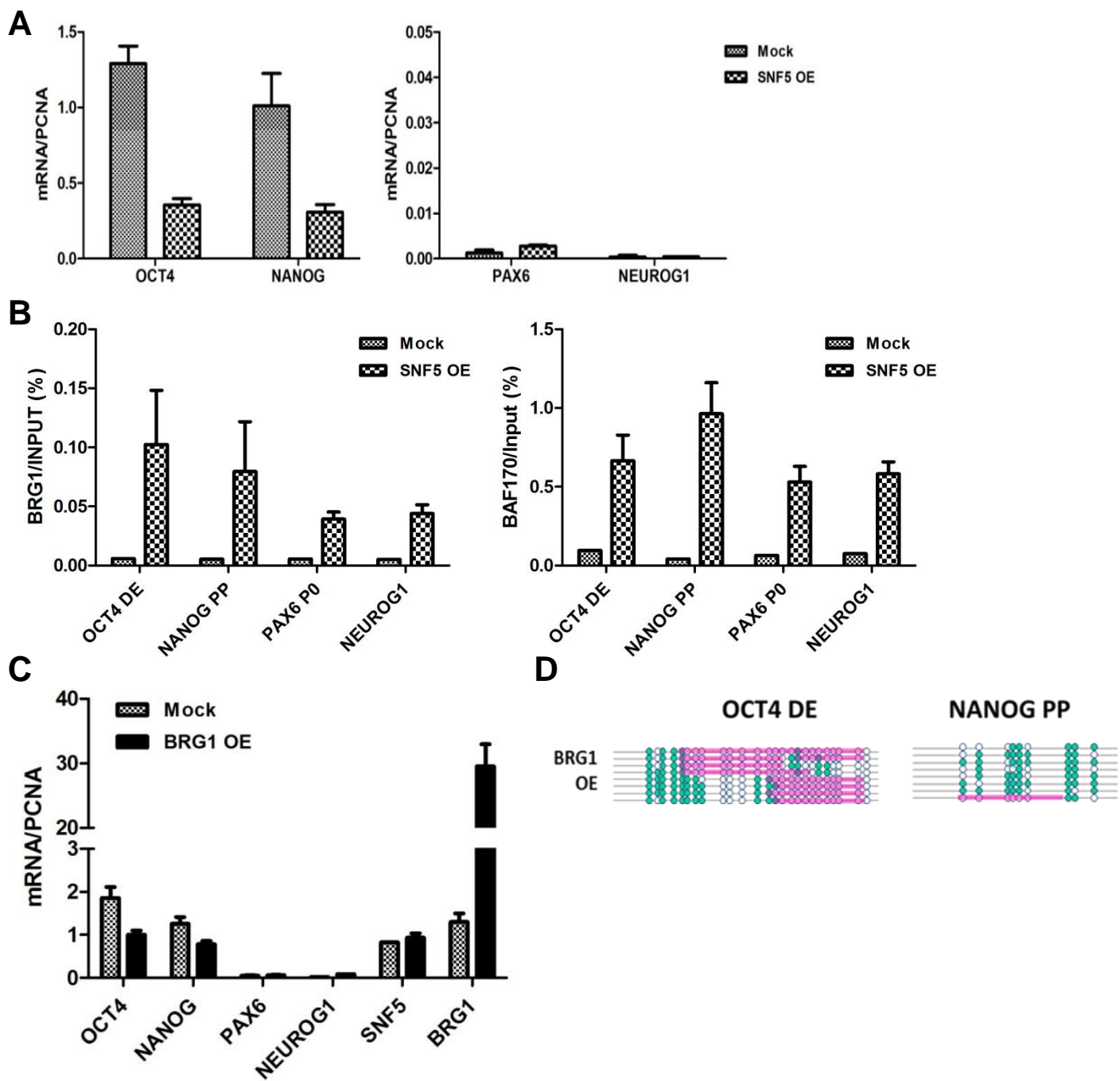

Supplement: Figure S4 — 72 h post-transfection with exogenous SNF5 overexpression vector in NCCIT cells, mRNA levels of OCT4, NANOG, PAX6 and NEUROG1 were analyzed by quantitative PCR (A). Quantitative PCR data were combined of two biological experiments. After SNF5 overexpression, BRG1 and BAF170 binding at the DNA regulatory regions of OCT4 target genes were analyzed by quantitative PCR (B). After BRG1 overexpression, OCT4 targets mRNA quantitative PCR (C) and NOMe-seq (D) performed. (PDF) [file pgen.1003459.s004.pdf]

**A**

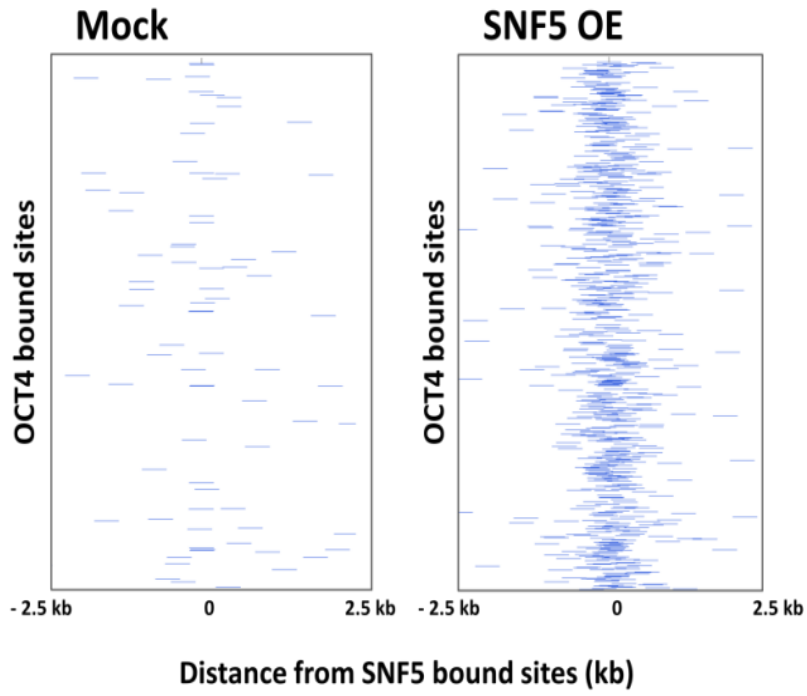

**B**

| Human                | Mouse | Brg1 bound genes | not Brg1 bound genes | All   |
|----------------------|-------|------------------|----------------------|-------|
| SNF5 bound genes     |       | 2400             | 4951                 | 7351  |
| not SNF5 bound genes |       | 1601             | 6626                 | 8227  |
| All                  |       | 4001             | 11577                | 15578 |

**C**

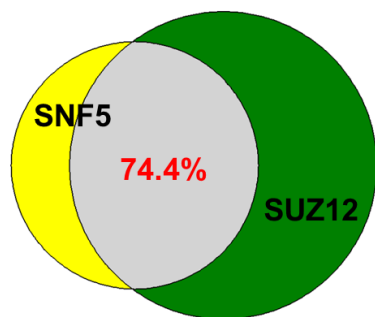

Supplement: Figure S5 — The binding plots show the localization of OCT4 bound sites relative to SNF5 bound sites (A). OCT4 bound sites (y axis) are displayed within a 5 kb window centered on the SNF5 bound site. Intensity at position 0 indicates that site overlap. Mouse Brg1 binding sites were downloaded from NCBI GSE14344 and compared with our SNF5 ChIP-seq data (B). SUZ12 binding sites (wgEncodeBroadHistoneH1hescSuz12051317Pk.broadPeak.gz) were downloaded from UCSC and compared with our SNF5 ChIP-seq data (C). By chi-square test, SNF5 bound genes and Suz12 bound genes significantly overlap with each other, with odds ratio = 2.71, and p-value<2.2×e-16. The Venn Diagram represented that 6736 (74.4%) were also bound by SUZ12 out of 9047 SNF5 bound genes. (PDF) [file pgen.1003459.s005.pdf]

**A**

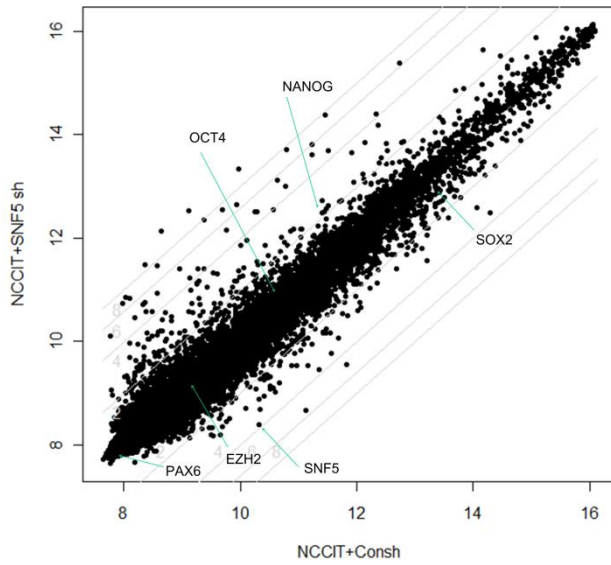

**B**

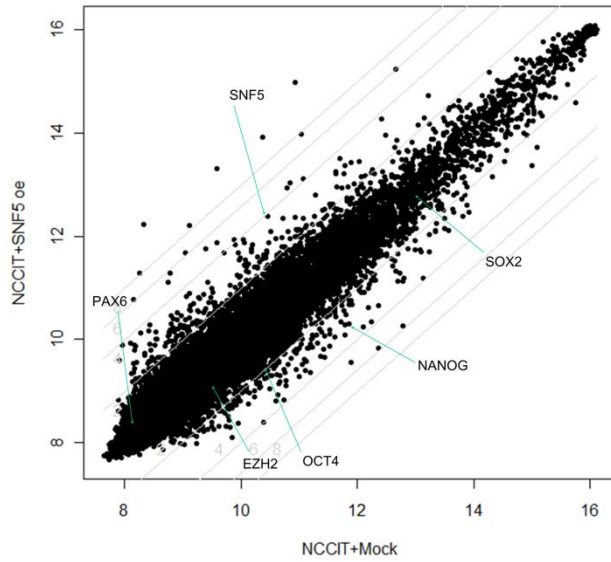

**C**

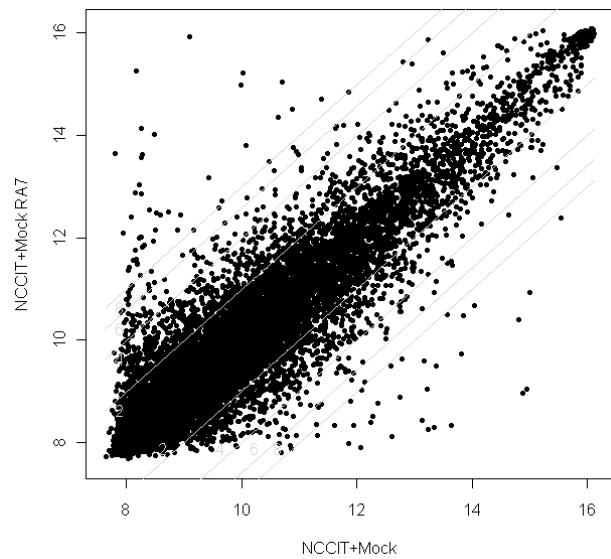

**D**

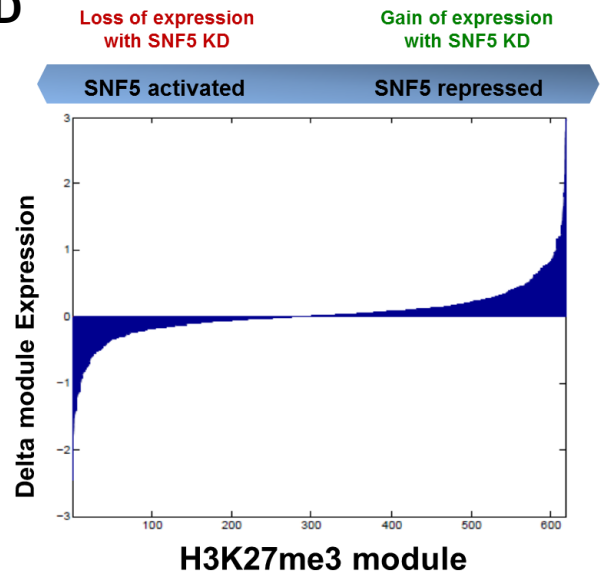

**E**

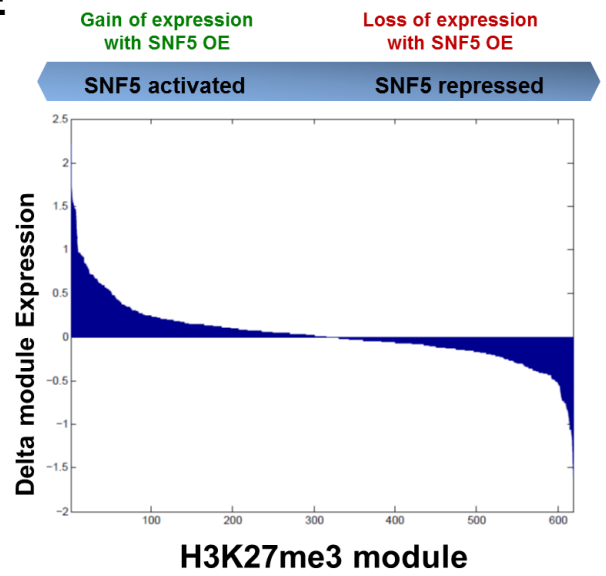

Supplement: Figure S6 — Scatter plots comparing global gene expression profiles between SNF5 shRNA #2 (shSNF5#2) and control shRNA (shControl) cell lines (A), between SNF5 overexpression (SNF5 OE) and Mock cell lines (B), and between RA treated and control NCCIT cells (C). Individual genes within the H3K27me3 bounded target gene module were analyzed in SNF5 knockdown (D) and overexpression (E) states. (PDF) [file pgen.1003459.s006.pdf]
